# From Hive to Highway: Waste Honeycombs as a Sustainable Modifier for Asphalt Binder Formulations in South Korea

**DOI:** 10.3390/ma16216934

**Published:** 2023-10-28

**Authors:** Nader Nciri, Namho Kim

**Affiliations:** 1 School of Industrial Design & Architectural Engineering, Korea University of Technology & Education, 1600 Chungjeol-ro, Byeongcheon-myeon, Dongnam-gu, Cheonan 31253, Chungnam, Republic of Korea; nader.nciri@koreatech.ac.kr; 2 School of Energy, Materials & Chemical Engineering, Korea University of Technology & Education, 1600 Chungjeol-ro, Byeongcheon-myeon, Dongnam-gu, Cheonan 31253, Chungnam, Republic of Korea

**Keywords:** waste honeycombs (WHCs), apiculture byproduct, asphalt binder formulations, SARA profile, asphaltene reduction, colloidal instability index (I_C_), SEM microstructure, binder behavior modification

## Abstract

Navigating the crossroads of sustainable infrastructure and innovative waste management, this research unveils the potential of waste honeycombs (WHCs)—an overlooked byproduct of apiculture—as a potent modifier for asphalt binder formulations. This endeavor addresses the dual challenge of enhancing road pavement sustainability and mitigating environmental degradation. A meticulous methodology evaluated the impact of varying WHC concentrations (5, 10, and 15 wt.%) on the asphalt binder, examining its attributes pre- and post-aging. Employing an array of analytical tools—thin-layer chromatography-flame ionization detection (TLC-FID); Fourier transform-infrared spectroscopy (FT-IR); scanning electron microscopy (SEM); thermogravimetric analysis (TGA); and a suite of conventional tests such as penetration, softening point, viscosity, ductility, dynamic shear rheometer (DSR), and multiple stress-creep recovery (MSCR)—provided a comprehensive insight into the binder’s behavior. TLC-FID analyses revealed that WHC, with its 92 wt.% resin content, altered the SARA profile across distinct aging conditions, notably reducing asphaltene content, a factor linked to binder stiffness. The colloidal instability index (I_C_) further attested to this, pointing to a more thermodynamically stable system with WHC’s inclusion. Meanwhile, FT-IR confirmed a physical interaction between WHC and asphalt without introducing new chemical entities. SEM observations highlighted the superior miscibility of WHC with asphalt, evidenced by a unique microtexture. With marked precision, TGA assessments unveiled a bolstering of asphalt’s inherent thermal resilience consequent to a minor WHC integration. From the conventional tests, shifts in penetration, softening point, and viscosity were observed, with reduced viscosity, indicating improved workability. Lastly, while rutting potential was sensitive to WHC concentrations, fatigue resistance notably heightened with minor to moderate WHC inclusions. In essence, this pioneering study advocates for WHC’s integration into asphalt formulations, offering enhanced road performance coupled with sustainable waste utilization. The findings underscore the synergy between environmental stewardship and infrastructural advancement.

## 1. Introduction

Over the past few decades, South Korea has undergone a dramatic transformation marked by rapid urbanization and explosive economic growth. Central to this change is the ever-growing need for efficient transportation infrastructure, particularly high-quality road pavements. Historically, the nation’s roadways, constructed primarily from conventional materials like asphalt and concrete, have held their ground. However, their vulnerabilities in the face of environmental extremes and rising vehicular demands cannot be downplayed. These factors lead to frequent maintenance, signifying an ongoing battle against wear and tear [1,2,3]. Parallel to this challenge, the construction sector grapples with issues such as resource scarcity and the complexities of waste management. These dual concerns highlight the urgent need for innovative, durable, yet sustainable solutions in road pavement construction. It is here that South Korea’s dedication to green urban development comes to the fore. 

The pursuit of alternative materials, especially those derived from waste, presents a chance for a groundbreaking change in infrastructure and environmental conservation. Waste honeycombs, a seemingly unlikely candidate, stand poised at the edge of this transformative opportunity. While South Korea boasts a modern urban fabric, it still contends with persistent road pavement issues that deeply affect its populace. Chief among the culprits are potholes, wear-induced fissures, inadequate drainage, and an uptick in vehicular movement due to urban growth [4,5]. Together, these issues present both monetary and societal challenges rooted in infrastructure wear, congestion, and road safety [4,5].

In response, South Korea, like many nations, is leaning into waste recycling for road construction. In our sustainable era, waste materials such as rubber [6], glass [7], and plastic [8] are finding renewed purpose in road pavements across the globe, extending their lifespans while combatting the environmental fallout of waste. A potential game changer in this arena is the often-overlooked beekeeping sector, producing honeycomb waste that might reshape road construction.

Honeycombs, composed largely of beeswax and an assortment of organic materials, are central to the bee ecosystem [9,10]. They offer storage and serve as breeding grounds. Predominantly inert, beeswax is resilient to water and many acids and possesses a melting point between 62 to 64 °C [9,10]. These qualities point to its potential as a road pavement additive. However, once these honeycombs are no longer in use, they often meet an undignified fate, discarded or burnt. Such actions exacerbate global waste concerns and release harmful compounds (e.g., antibiotics, antifungals, pesticides, Varroa mite control agents, hive cleaners and disinfectants, and so forth) [11,12]. But within this waste lies a golden opportunity for sustainable, environmentally friendly infrastructure.

The path from waste honeycomb to viable material involves beeswax extraction, material granulation, and integration with asphalt. Due to its chemical composition—dominated by hydrocarbons accounting for 15 wt.%, esters forming 71 wt.%, free acids at 8 wt.%, and other compounds making up the remaining 6 wt.% [13]—honeycomb can act as a formidable adhesive with asphalt. Melding recycled honeycombs into road construction can result in reduced environmental impact, increased road durability, and a green future for South Korea, bridging world-class infrastructure with environmental responsibility.

Yet, the full potential of honeycombs can only be realized when juxtaposed against their negative impact as waste. Repurposing this waste for road pavement mitigates its environmental threats, fostering a balance between green development and waste management. Honeycombs’ unique properties, beyond waste reduction, offer an array of benefits. Their beeswax content, inert and highly pliable, provides robustness and safeguards against damage [14,15,16]. Coupled with other bee-derived materials (e.g., pollen oils and propolis/bee glue) known for their antibacterial properties [14,15,16], honeycombs emerge as robust and lasting, apt for infrastructure projects.

Waste honeycombs, sharing similarities with asphalt compounds, offer a unique opportunity in road pavement techniques. Their particular attributes, coupled with environmental benefits, deserve crucial attention in modern pavement paradigms. Integrating honeycombs with asphalt can reduce reliance on petroleum-based products, emphasizing eco-friendly binders. This approach, addressing the challenges posed by beekeeping waste, intensifies the push for sustainable infrastructure. South Korea’s alignment with this innovative method showcases its commitment to sustainable goals and a circular economy, setting a global benchmark for blending infrastructural advancement with environmental conservation.

In summation, South Korea’s infrastructure landscape may stand to benefit profoundly from the integration of waste honeycombs—a sustainable asset yet to be fully tapped. The crux of this study is to comprehensively decipher the potential of these honeycombs as pivotal modifiers or additives in asphalt formulations. By employing a meticulous array of chemical, physical, and rheological assessments, we aim to delineate the nuanced impacts of honeycomb integration on pavement characteristics. While the complete ramifications remain under investigation, this research endeavors to establish a paradigm for future eco-conscious, innovative road construction techniques.

## 2. Materials and Methods

### 2.1. Extraction and Purification Procedure of Beeswax from Honeycomb Residuals

Derived from regional apiaries, honeycomb residuals underwent an advanced preparatory phase [17]. Initially, these materials were subjected to a meticulous multistage cleansing protocol. Repeated washings were executed to methodically expunge impurities, contaminants, remnants of deceased bees and larvae, and any vestigial saccharides.

Subsequent to this rigorous purification, the honeycombs were systematically fragmented into uniformly dimensioned particulates, each measuring approximately 0.5 cm × 0.8 cm × 0.2 cm. These calibrated fragments were then introduced into an aluminum containment vessel.

For optimal uniformity, the fragmented particulates were interfused with distilled water, initiating a controlled amalgamation. A thermostatic plate was deployed, ensuring the consistent phase transition of the beeswax within a tepid aqueous medium. Upon attaining a homogenized liquid state, the concoction was sequestered, subjecting it to a 24-h thermodynamic equilibrium process culminating in its solidification.

Post-solidification, the resulting beeswax matrix was judiciously transferred into a borosilicate containment apparatus. Precision heating was applied using an oven dialed to a stringent 80 °C calibration. The material was sustained at this thermic specification for 2 h to remove trace moisture. Subsequent to this thermic phase, the liquified beeswax was channeled through a fine-meshed cheesecloth, a step vital to the stratification and attainment of the purest beeswax, ensuring the absolute segregation from residual honeycomb detritus.

**Figure 1:**
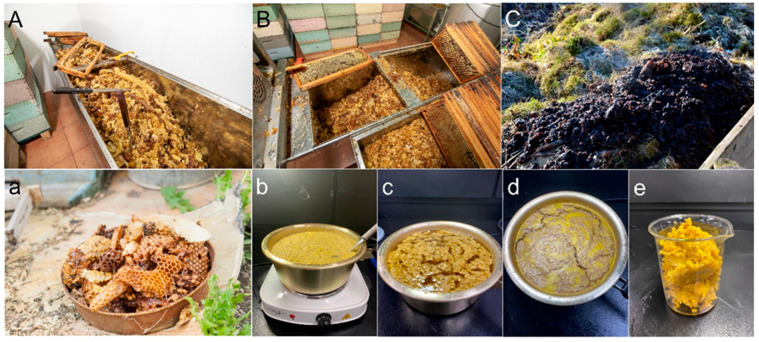
Detailed portrayal of honeycomb waste management and beeswax extraction. (**A**–**C**) Depictions of residual honeycombs post-harvest, which are typically discarded in open terrain. (**a**) Collection of discarded honeycombs. (**b**) Controlled heating of honeycomb residues in distilled water on a heating plate, facilitating the melting phase of beeswax. (**c**) Observation of beeswax transition to a liquid state atop the warm distilled water. (**d**) Subsequent crystallization of the beeswax on the distilled water surface after an overnight period. (**e**) Rigorous extraction and refinement process of beeswax: recovery from the aqueous layer followed by a dehydration phase in an oven set at 80 °C for 2 h and a final filtration through fine-meshed cheesecloth to ensure purity.

The conclusion of this protocol was the derivation of beeswax exemplifying unparalleled purity, priming it for specialized applications, such as its integration within asphalt binder matrices. The comprehensive procedural architecture is illustrated in Figure 1.

### 2.2. Chemical-Physical Characteristics of Waste Honeycomb (WHC)

#### 2.2.1. Elemental and Physicochemical Profile of WHC

As delineated in Table 1, the elemental composition of the waste honeycomb (WHC) was studied meticulously. The findings were as follows: Carbon constituted a significant proportion of the honeycomb, with a weight percentage of 82.15 ± 0.20 wt.%. Hydrogen followed suit, registering 14.09 ± 0.04 wt.%, while oxygen was found in a lesser amount of 3.92 ± 0.02 wt.%. Interestingly, nitrogen was found in a minuscule proportion (0.02 ± 0.02 wt.%), and sulfur was virtually undetectable, amounting to 0.00 wt.%. Delving deeper, the SARA generic fractions of the WHC were dissected. The predominant fraction was resins, amounting to 92.07 ± 0.89 wt.%, whereas saturates were present at 7.92 ± 0.89 wt.%. Both aromatics and asphaltenes were non-existent, with their weight percentages being a flat 0.00 wt.%. Moving onto the physical attributes of the WHC, the softening point or slip melting point was recorded at 34 °C, a standard measure for such materials. At an elevated temperature of 100 °C, its rotational viscosity was observed to be 4.70 ± 0.00 cP. Furthermore, the melting point of the WHC lies in the range of 64 °C. A notable trait of the WHC is its solubility; it seamlessly dissolves in organic solvents such as *n*-hexane, toluene, dichloromethane, and so on. The density of the WHC at room temperature (viz., 25 °C) stands at 0.9500 g cm^−3^. Visually, the waste honeycomb boasts a golden hue, and olfactorily, it exudes a sweet to mild aroma (Figure 1e).

#### 2.2.2. XRD Insights into WHC’s Beeswax Composition

X-ray diffraction (XRD) was employed to discern the crystalline patterns present in the waste honeycomb (WHC). The analysis harnessed the capabilities of the Bruker AXS D8 Advance Diffractometer, sourced from Bruker AXS GmbH, Karlsruhe, Germany. This instrument operated using a Cu-Kα radiation source with a wavelength (λ) of 1.54005 Å, maintained at a voltage setting of 40 kV and a current flow of 40 mA. The subsequent diffraction profiles were captured over a 2θ range of 10° to 90°, progressing at a steady pace of 1° per min.

**Figure 2:**
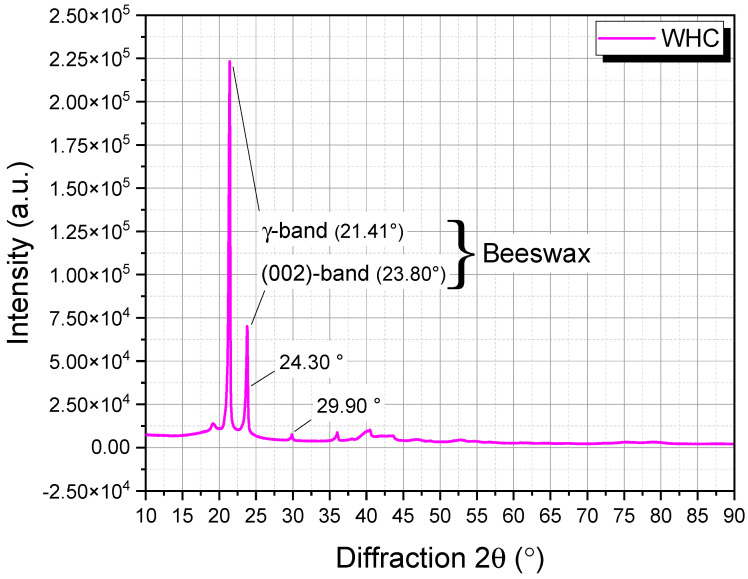
XRD spectrum of waste honeycomb (WHC).

The WHC’s XRD pattern, prominently displayed in Figure 2, predominantly reflects the complex molecular signatures of beeswax. Renowned for its elaborate constitution, beeswax encompasses a vast array of constituents, such as esters, hydrocarbons, free acids, and alcohols. This rich tapestry of components manifests as both crystalline and amorphous phases in its XRD profile.

At the heart of this spectrum, a characteristic peak stands pronounced at approximately 21.41° (2θ). This peak or γ-band serves as an emblematic representation of the crystalline nature of methylene chains within beeswax [18]. Further, the region between 20–25° (2θ) reveals a broad and diffused peak, capturing the amorphous elements of the wax [18]. Augmenting this are distinct crystalline peaks observed at 23.80° (002-band), 24.30°, and 29.90° (2θ), which are strongly affiliated with beeswax’s inherent monohydrate form [18].

Beyond these major spectral landmarks, one can also discern subtle ancillary peaks, indicative of nuanced components. These may encompass entities such as cerotic acid, a spectrum of hydrocarbons, and long-chain esters [18], further underscoring the multi-dimensional character of beeswax within the WHC.

The interpretation of these peak positions and their respective intensities is vital. Factors such as geographical origin, purification methods, and potential introduction of extraneous materials can introduce variations in the XRD profile. Such complexities, particularly in the context of waste honeycombs intended for asphalt modification, underline the importance of a comprehensive characterization. While the XRD provides pivotal insights, supplementing this with methodologies like thermogravimetric analysis (TGA), Fourier-transform infrared spectroscopy (FTIR), and scanning electron microscopy (SEM) can grant a more complete comprehension of the waste honeycombs’ potential in asphalt applications.

### 2.3. Formulation of Asphalt Mixtures Incorporating Waste Honeycomb (WHC)

The bedrock of this scientific exploration was an AP-5 asphalt (PG 70–10) variant generously made available by the South Korean Federation of Ascon Industry Cooperative R&D Center based in Osan-si, Gyeonggi-do, Republic of Korea. This asphalt’s physicochemical characteristics, forming the foundation of our analysis, are meticulously presented in Table 2.

Our study leveraged the potential of waste honeycombs (WHCs) responsibly sourced from a domestic honey production establishment located in Cheonan, South Korea, visually represented in Figure 1e. An elaborate account of the intriguing chemical and physical traits of these WHCs is provided in Table 1.

The experimental methodology primarily involved a high-performance Silverson L5M-A mixer (Model No. GLHMD-B100, Silverson, East Longmeadow, MA, USA) operating at a rotational speed of 3000 rpm in tandem with a high-precision heating mantle (Model No. GLHMD-B100 from Global Lab Co., Ltd., Siheung-si, Republic of Korea) maintaining a stable thermal environment at 180 °C for the production of WHC-infused asphalt concoctions [19,20,21].

The benchmark binder underwent a controlled thermal regimen in an oven at 140 °C for an uninterrupted span of 2 h. This pivotal stage was engineered to secure the optimal rheological state of the binder during the blending operation, fostering homogeneity in the final blend whilst simultaneously curtailing the likelihood of oxidative disintegration.

The subsequent composite blends were methodically prepared by allocating approximately 600 g of molten asphalt into 1000 mL cylindrical aluminum vessels followed by an incremental heat treatment from 140 to 175 °C [19,20,21]. Subsequent to this stage, varying quantities of WHCs were cautiously integrated into the binder, ranging between 5, 10, and 15 wt.% of the total blend’s weight. This approach catered to potential unknowns related to the initial composition, binder reactivity, and thermal behavior of the WHCs.

The comprehensive assessment of the blends’ physicochemical, microstructural, thermo-morphological, and rheological attributes necessitated the strategic selection of wide concentration gradients. Upon deriving insightful data from this meticulous exploration, the ideal WHC concentration to attain premium engineering properties could be conclusively determined.

After maintaining the blend at 180 °C for a duration of 2 h [19,20,21], both the unaltered and WHC-incorporated asphalt samples displayed satisfactory consistency. Once the formulation stage was concluded, the bituminous specimens were securely transferred into compact metal receptacles, sealed hermetically, and kept at room temperature (approximately 25 °C) in anticipation of future artificial weathering experiments and performance evaluations.

### 2.4. Rigorous Laboratory Aging Protocols for Asphalt: Exploring the Rheological and Physicochemical Transformations

The crux of this study lies in the precise simulation and evaluation of the aging processes in AP-5 asphalt with and without the incorporation of waste honeycombs (WHCs) at concentrations of 5, 10, and 15 wt.%. Utilizing the sophisticated laboratory techniques of a rolling thin-film oven (RTFO) and a pressure aging vessel (PAV), the samples were subjected to artificial aging processes, paving the way for detailed physicochemical and rheological analyses.

#### 2.4.1. Short-Term Aging Simulation of Asphalt Binders Using the Rolling Thin-Film Oven (RTFO) Method as per ASTM D8272-19

The rolling thin-film oven (RTFO) method was employed to mimic short-term aging in line with ASTM D8272-19 [22]. Here, eight carefully measured fluid binder samples, each weighing 35 ± 0.50 g, were exposed to an environment of 163 ± 0.50 °C for 85 min under a consistent airflow rate of 4000 mL min^−1^. This strategy effectively reproduces the oxidative aging that asphalt undergoes during the production, transportation, and installation phases, thus providing valuable insights into the binder’s resilience and integrity in response to operational stresses.

#### 2.4.2. Assessment of Binder’s Long-Term Aging Using a Pressure Aging Vessel (PAV) in Line with ASTM D6521-13

Pressure aging vessel (PAV) aging, representative of long-term aging, was diligently performed in accordance with ASTM D6521-13 [23]. RTFO-treated binder samples, each weighing 50 ± 0.50 g, were introduced into stainless steel pans and subsequently exposed to the stringent conditions of a PAV apparatus (PAV3, Applied Test Systems LLC, Butler, PA, USA). The procedure, which exposes the samples to a combination of 100 °C temperature and 2.10 MPa pressure over a 20-h duration, faithfully replicates the aging experienced over five to ten years in service, offering a credible forecast of the binder’s long-term performance characteristics.

#### 2.4.3. Degassing Protocol for Asphalt Samples following PAV Aging Using a VDO 81-PV2610 Vacuum Degassing Oven

Following the PAV aging process, the asphalt samples were subjected to a degassing protocol for 30 min at 170 °C executed in a VDO 81-PV2610 Vacuum Degassing Oven (NOVA Measurements LLC, Atlixco, México). This procedure ensured the elimination of any trapped air pockets that might have been introduced during the PAV aging, thereby ensuring the binder’s homogeneity and enhancing its consistency for subsequent testing.

Upon concluding the aging procedures, the bitumen specimens subjected to short-term and long-term aging were meticulously stored in sealed metal containers, mitigating the risk of contamination or potential degradation. These carefully preserved samples were then primed for subsequent expedited testing protocols.

This methodical and comprehensive approach fosters a deep understanding of the inherent physicochemical and rheological modifications induced in asphalt binders under simulated aging conditions.

### 2.5. Characterization and Quantitative Analysis of Waste Honeycomb-Infused Asphalt Using the TLC-FID System: A Comprehensive Experimental Procedure

The key instrument used was the TLC-FID system (Iatron Laboratories, Tokyo, Japan) with a metallic rack and Chromarod-S5 silica rods (LSI Medience Corporation, Tokyo, Japan). This system analyzed the effects of different waste honeycomb (WHC) proportions (5, 10, and 15 wt.%) on the base AP-5 asphalt’s chemical makeup in both its original and aged states.

Chromarods of 15.2 cm in length with a 5 µm particle size and a 60 Å pore diameter were purified using a hydrogen flame from the FID. This step ensured high data accuracy by removing impurities. The detector utilized hydrogen gas and air at flow rates of 160 mL min^−1^ and 2 L min^−1^, respectively. A 2% sample solution of bitumen/dichloromethane or WHC/dichloromethane was used, with 1 µL applied onto the chromarod using a 5 µL Drummond microdispenser (Drummond Scientific, Broomall, PA, USA) [24].

The chromarod underwent saturation in three solvents: *n*-hexane, toluene, and a methanol/dichloromethane mix. In the TLC process, asphaltenes remained stationary while other components like saturates, aromatics, and resins were eluted. Igniting these components with a hydrogen flame yielded organic ions, which were detected by the FID [24]. The SARA (i.e., saturates, aromatics, resins, and asphaltenes) content was quantified from the FID readings. Post-immersion, rods were dried at 85 °C for about 2 min [24].

For data reliability, five samples were analyzed for each WHC–asphalt mixture, and each sample underwent Iatroscan analysis four times, ensuring accurate results [24].

### 2.6. Comprehensive FT-IR Spectroscopic Analysis of WHC-Treated Asphalt: Methodology, Instrumentation, and Implications on Chemical Structure Pre- and Post-Aging

An in-depth Fourier-transform infrared spectroscopy (FT-IR) study was conducted on the WHC and WHC-treated samples. FT-IR is utilized for its ability to assess a material’s chemical structure, especially for characterizing molecular functional groups. Using the Hyperion 3000 FT-IR Spectrometer (Bruker Optics, Ettlinger, Germany) with a spectral resolution of 1 cm^−1^, data were acquired over a wavenumber range of 4000 to 650 cm^−1^. Each sample underwent an average of 30 scans to ensure a reliable signal-to-noise ratio. For optimal detection, samples were prepared with Potassium Bromide (KBr) due to its infrared transparency. This study aimed to evaluate the influence of waste honeycomb concentrations (5, 10, and 15 wt.% WHC) on AP-5 asphalt’s chemical structure, comparing pre- and post-aging.

### 2.7. Scanning Electron Microscopy (SEM) Analysis of Waste Honeycomb-Infused AP-5 Bitumen: Insights into Surface Morphology and Optimization of Sample Preparation Techniques

The JSM-6010LA scanning electron microscope (SEM, JEOL Ltd., Tokyo, Japan) was used to assess the effects of waste honeycomb (WHC) concentrations (5, 10, and 15 wt.%) on the microsurface and microtexture properties of unaged/plain AP-5 base bitumen. To enhance electrical conductivities for SEM, samples were immersed in liquid nitrogen (LN_2_) at −80 °C. Post-cryogenic treatment, they were coated with a thin layer of gold using a precision X sputter coater (Sputter Coater Model 108auto C3783, Cressington Scientific Instruments, Watford, UK) to improve imaging quality. SEM micrographs were taken under specified parameters: ×3000 magnification, 5 nA beam current, 10 nm working distance, and 5 kV accelerating voltage to capture detailed morphological features.

### 2.8. Thermogravimetric Analysis (TGA) of Waste Honeycomb-Infused AP-5 Bitumen: Assessing Thermal Stability, Degradation Kinetics, and Variability in Thermal Behavior

Thermogravimetric analysis (TGA) was employed to study the thermal behavior of the samples, offering insights into their thermal stability and degradation patterns. The TGA Q500 from TA Instruments (New Castle, DE, USA) was chosen for its high resolution and accuracy. This study examined the impact of varying waste honeycombs ratios (5, 10, and 15 wt.% WHC) on the thermal attributes of fresh/virgin AP-5 bitumen. Samples weighing 10–15 mg were heated from 25 °C to 1000 °C at a rate of 20 °C min^−1^ under a nitrogen atmosphere. The TGA procedure was repeated three times per sample to ensure consistent results. 

### 2.9. Morphological and Rheological Evaluation of Waste Honeycomb-Integrated AP-5 Asphalt: A Comprehensive Analysis Based on ASTM Standards and Laboratory Tests

The exploration presented here examines the intricate implications of integrating waste honeycombs (WHCs) at proportions of 5, 10, and 15 wt.% into the matrix of AP-5 asphalt cement. The resultant morphological and rheological modifications to the asphalt were rigorously analyzed through a variety of laboratory-based investigations. This included penetration, softening point, viscosity, and ductility tests executed in strict conformance with several ASTM standards.

#### 2.9.1. Penetration

In the assessment of hardness or softness—a pivotal characteristic dictating asphalt performance—the penetration test was conducted following the procedural guidelines of ASTM D5 [25]. Employing a Humboldt Mfg Electric Penetrometer (Humboldt Mfg. Co., Elgin, IL, USA), this method entailed the vertical immersion of a 100 g loaded standard needle into a temperature-controlled asphalt sample at 25 °C for a five-second duration. The extent of needle penetration, gauged with a precision of 1/10 mm, furnished reliable estimates of the binder’s hardness. The penetration test was conducted five times to ensure reproducibility, accuracy, and methodological reliability.

#### 2.9.2. Softening Point

Subsequently, the thermal behavior of the binder, marked by its softening point, was evaluated using the Ring and Ball Test Apparatus RKA 5 (Anton Paar GmbH, Ashland, VA, USA) in accordance with ASTM D36 [26]. A bituminous disk was subjected to a downward pressure exerted by a 1 cm diameter steel ball, and its flow behavior was observed over a span of 2.5 cm. The temperature at which the binder attained a predefined flow rate was identified as the softening point (T_R&B_), thereby encapsulating its thermal stability and resistance to deformation. The ring and ball assay was performed on four distinct occasions to ascertain its reproducibility, ensure analytical accuracy, and validate its methodological robustness.

#### 2.9.3. Rotational Viscosity

Rotational viscosity (RV), a cardinal rheological property, was determined using a Brookfield DV III Rheometer (Brookfield, Middleboro, MA, USA) following the precise guidelines of ASTM D4402 [27]. At a constant temperature of 135 °C, the RV was established by quantifying the torque necessary to maintain a SC4-27 spindle at 20 rpm within a controlled quantity of asphalt (10 ± 0.50 g). This parameter quantifies the internal friction or resistance to deformation, elucidating vital attributes about the asphalt’s behavior during processes such as mixing, compaction, and transportation. The rotational viscosity assessment was undertaken on four distinct occasions, emphasizing a commitment to ensuring reproducibility, upholding analytical precision, and validating the robustness of the methodology.

#### 2.9.4. Ductility

The ductility test, an intrinsic analysis of the binder’s elasticity and adhesive potential, was executed at 25 °C using a ductilometer apparatus (Woojin Precision Co., Ltd., Gwangju-si, Republic of Korea) in strict accordance with the ASTM D113 [28] protocol. This test involves elongating a standard-sized briquette of binder at a fixed rate of 5 cm per minute within a water bath. The elongation potential before rupture effectively measures the asphalt’s ductility, a critical parameter demonstrating the binder’s ability to withstand deformation without failure. The stretching test was meticulously carried out on four separate occasions to ascertain its reproducibility, guarantee its accuracy, and ensure the overall reliability of the results obtained.

This sophisticated suite of assessments provides a robust profile of the morphological and rheological modifications induced in asphalt incorporated with WHC. 

### 2.10. Advanced Rheological Analysis of AP-5 Asphalt Cement with Varied WHC Proportions Using the Dynamic Shear Rheometer (DSR)

This study utilized the dynamic shear rheometer (DSR) from Thermo Fisher Scientific (Newington, NH, USA) to investigate the impact of different waste honeycomb (WHC) concentrations (5, 10, 15 wt.%) on the viscoelastic properties of AP-5 asphalt cement. Adhering to ASTM D7175 [29], the DSR testing focused on asphalt’s rutting and fatigue cracking behaviors within temperature ranges of 46–82 °C and 4–40 °C, respectively. Samples underwent testing at a loading frequency of 10 rad s^−1^ (1.59 Hz), mimicking asphalt’s shear stress under traffic speeds of 55 mph (90 km h^−1^). Data-derived rutting and fatigue cracking factors, G*/sin δ and G*.sin δ, used the phase angle (δ) and complex shear modulus |G*|. For temperatures of 4–40 °C, a 2 mm thick and 8 mm diameter specimen was used, while a 1 mm thick and 25 mm diameter specimen was used for 46–82 °C. This methodology offers insights into asphalt’s performance under varied WHC proportions, advancing knowledge for improved road infrastructure.

### 2.11. Comprehensive Rheological Assessment of AP-5 Bitumen with WHC Incorporation: Leveraging the Multiple Stress Creep Recovery (MSCR) Methodology for Rutting Susceptibility Analysis

This study aimed to analyze the basic rheological properties of bituminous binders and understand the impact of waste honeycomb (WHC) on the rutting resistance of AP-5 bitumen using the multiple stress creep recovery (MSCR) test. The test was conducted in compliance with the AASHTO T 350-19 protocol [30] using a dynamic shear rheometer (DSR) from ThermoFisher (Thermo Scientific™ HAAKE™ MARS™ Rheometer, Thermo Fisher Scientific, Newington, NH, USA).

The MSCR test, simulating real-world traffic loading and unloading conditions, was performed at 64 °C, representing the average 7-day pavement design temperature in South Korea. A 25 mm diameter, 1 mm thick RTFO-aged asphalt sample was examined, undergoing ten consecutive loading and recovery cycles with stress levels of 0.1 and 3.2 kPa. Each cycle consisted of a 1 s loading stage followed by a 9 s recovery phase.

Key metrics derived post-test included percent recovery (*R*%) and the non-recoverable creep compliance (*J_nr_*), an index indicating rutting potential. The *J_nr_* metric highlights the permanent deformation in the sample after multiple creep–recovery cycles relative to the applied stress, offering insights into the binder’s deformative reactions. The *R*% measures the binder’s elasticity and its response to stress.

Important equations utilized were:

(1) $$J_{nr\;0.1}\left(\frac{1}{kPa}\right)=\frac{1}{10}\left\{\sum_{n=1}^{10}\left(\frac{Non-Recoverable\;Strain}{0.1}\right)\right\}$$

(2) $$J_{nr\;3.2}\left(\frac{1}{kPa}\right)=\frac{1}{10}\left\{\sum_{n=1}^{10}\left(\frac{Non-Recoverable\;Strain}{3.2}\right)\right\}$$

(3) $${∆\;J}_{nr\;}\left(\%\right)=\left\{\left(\frac{J_{nr\;3.2}-J_{nr\;0.1}}{J_{nr\;0.1}}\right)\times 100\right\}\;\leq 75\%$$

(4) $$R\;\left(\%\right)=\frac{1}{10}\left\{\sum_{n=1}^{10}\left(\frac{Recoverable\;Strain}{Peak\;Strain}\right)\times 100\right\}$$

The results from the MSCR test provide crucial insights into the bituminous binder’s performance and its ability to withstand rutting from varying traffic and environmental conditions.

## 3. Results and Discussion

### 3.1. In-Depth Analysis of SARA Component Dynamics in Asphalt Binders Incorporating Waste Honeycomb (WHC): An Exploration in Asphalt Colloidal Science

In the sophisticated domain of petroleum product characterization, the method of thin-layer chromatography-flame ionization detection (TLC-FID), also known as Iatroscan, stands as a paramount analytical tool. Its intrinsic accuracy, rapidity, and streamlined approach facilitate the comprehensive breakdown of materials, including bitumen, heavy oils, and shale oils, into four cardinal fractions: Saturates (S), Aromatics (A), Resins (R), and Asphaltenes (A)—collectively termed the SARA fractions. This fractionation is orchestrated based on the pronounced disparities in polarizability and polarity amongst these fractions, obviating the requirement for initial asphaltene precipitation [31].

#### 3.1.1. Compositional Dynamics in Pristine AP-5 Bitumen and Waste Honeycomb (WHC)

**Figure 3:**
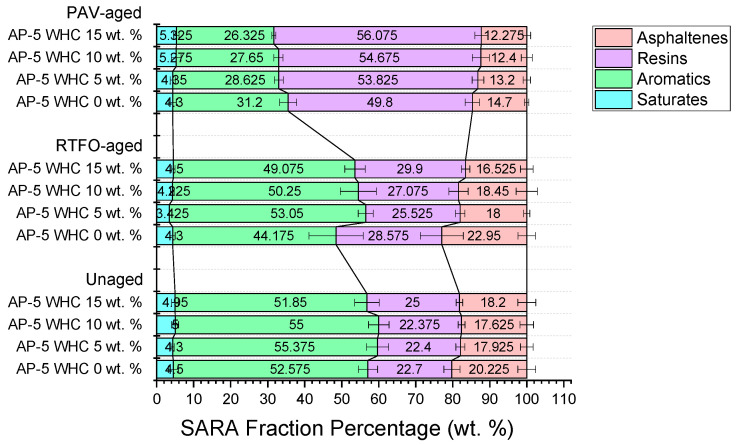
Influence of differential concentrations of waste honeycomb (5, 10, 15 wt.% WHC) on the foundational SARA fractions of base AP-5 asphalt prior to and subsequent to RTFO and PAV aging processes.

The central aim of the Iatroscan study revolves around discerning the functional interplay between the fractional composition of unadulterated AP-5 bitumen and waste honeycomb (WHC) both pre-aging and post-aging. Figure 3 delineates that raw AP-5 asphalt is characterized by a pronounced aromatic presence (52.57 ± 2.58 wt.%), an intermediate resin content (22.70 ± 2.18 wt.%), a reduced asphaltene concentration (20.22 ± 2.40 wt.%), and minimal saturate inclusion (4.50 ± 1.32 wt.%). Contrastingly, WHC is characterized by a pronounced resinous profile, constituting approximately 92.07 ± 0.89 wt.%. This composition intricately encompasses molecules such as esters, free fatty acids, and fatty alcohols, with trace constituents of other compounds. The saturate fraction within WHC is notably delineated at an estimated 7.92 ± 0.89 wt.%, predominantly comprised of *n*-alkanes.

#### 3.1.2. SARA Fraction Adjustments with WHC Integration in Unaged Conditions

Incorporating waste honeycomb (WHC) into unaged asphalt leads to distinct alterations in the SARA fractions, as illustrated by Figure 3. Predominantly composed of resins (92.07 ± 0.89 wt.%), WHC also contributes a smaller fraction of saturates (7.92 ± 0.89 wt.%) to the mix. This contribution, complemented by WHC’s inherent molecular elements such as free fatty alcohols, results in an uptick in the asphalt’s saturate levels.

The aromatic fraction in the asphalt also sees an enhancement. Some molecular constituents present in WHC, including specific esters and free fatty alcohols, might engage in stabilizing interactions with the asphalt’s compounds, thus fostering this increase in aromatics.

The resin content in the asphalt notably surges with the continuous integration of WHC, a direct outcome of WHC’s dominant resin component. This infusion of resins is crucial, as resins in asphalt play a pivotal role in ensuring asphaltenes are well solubilized and uniformly dispersed.

As a corollary, the asphaltene faction undergoes modulation. Given WHC’s resin-rich composition, asphaltenes achieve a better distribution within the matrix, reflecting as a perceived reduction in asphaltene concentration. This altered profile in the unaged scenario underscores the interactivity between WHC’s molecular makeup and the native components of the asphalt.

Given the intricate nature of waste honeycombs, it is not purely a uni-resinous entity. Instead, it is a sophisticated composite material encompassing diverse constituents. These include beeswax, esters, free fatty acids, alcohols, and residues of honey and pollen, among others. When integrated with asphalt in varying ratios, this multifaceted composition has the potential to influence the distribution of its SARA components, namely saturates, aromatics, resins, and asphaltenes. Moreover, each individual component of the honeycomb can engage in distinct physiochemical interactions with the binder elements, both in unaged and aged conditions. Consequently, these myriad interactions culminate in the SARA profile exhibiting a non-linear variation trend.

#### 3.1.3. SARA Fraction Dynamics under Short-Term Aging (RTFO)

As evidenced by Figure 3, upon RTFO aging, the introduction of waste honeycomb (WHC) into the AP-5 asphalt matrix resulted in distinctive shifts within the SARA fractions, particularly in the aromatic and resin components. This transformative behavior can be mapped back to the WHC’s significant resinous character, boasting 92.07 ± 0.89 wt.%. The escalation in aromatic content can be deciphered through a dual lens: the inherent oxidative aging mechanism of RTFO fosters polymerization of lighter molecules, integrating them into the aromatic fraction [32], and there is dynamic interplay between the asphalt matrix and WHC’s components, such as esters and free fatty alcohols. This interaction, coupled with the influence of residual honey components in WHC, amplifies the aromatic presence. Furthermore, the decline in asphaltenes owes its dynamics to the peptizing potency of maltenes, an effect accentuated by WHC’s rich resin contribution, ensuring a well-dispersed state of asphaltenes. Conclusively, the post-RTFO SARA fraction alterations are a confluence of WHC’s compositional fingerprint, intrinsic asphalt aging mechanisms, and the synergistic interactions between asphalt constituents and WHC residues.

#### 3.1.4. SARA Fraction Dynamics under Long-Term Aging (PAV)

Figure 3 demonstrates that during prolonged PAV aging, the integration of waste honeycomb (WHC) into the asphalt matrix induces palpable shifts in the SARA fractions. A marked increase in the saturates and resins fractions is evident, accompanied by a significant decline in the concentrations of both aromatic and asphaltene components.

WHC’s intrinsic composition, primarily composed of resins (92.07 ± 0.82 wt.%) with a minor portion of saturates (7.92 ± 0.89 wt.%), directly contributes to the enhanced saturates and resins fractions in the asphalt mixture upon its addition. This straightforward addition is only one facet of the observed change; the natural aging progression also plays a pivotal role. As the aging ensues, certain molecules in the aromatic phase undergo a transition, progressively converting into the resin phase [33]. This mechanistic shift not only amplifies the resin concentration but also leads to the concomitant decrease in the aromatic fraction [33].

Furthermore, as the aging process intensifies, the transformation of resins to asphaltenes becomes more pronounced [33]. However, the dominant resinous character of WHC, supplemented by the potential interactions of its honey residues, exerts a pronounced peptizing effect. This effect acts as a countermeasure, reducing the propensity for resin molecules to evolve into larger asphaltene conglomerates.

In more refined terms, one might postulate that the pronounced augmentation in resin content during PAV aging, whether subsequent to the systematic incorporation of waste honeycomb (WHC) into the asphalt binder or not, has the potential to disrupt the thermodynamic stability of the binder system. This intensification in resin concentration may reach a threshold at which these molecules begin to infiltrate the micellar structure of asphaltenes. Upon doing so, they could induce fragmentation, resulting in numerous resin–asphaltene aggregates that are predominantly resin-rich. Such a phenomenon would invariably lead to a consistent elevation in resin content while concurrently diminishing the proportion of asphaltenes. However, it is imperative to note that this hypothesis, while plausible, requires further elucidation and rigorous investigation to be fully validated.

To conclude, the dynamics witnessed in the SARA fractions during PAV aging are sculpted by a combination of WHC’s unique compositional attributes, the inherent molecular transitions associated with asphalt aging, and the modulating influence of the peptizing effect, which mitigate the expected increase in asphaltene content.

#### 3.1.5. Conclusive Insights

The integration of waste honeycomb (WHC) into asphalt presents significant advancements for road construction. Asphalt’s performance, influenced by its molecular makeup, sees marked improvements with WHC inclusion. The resulting asphalt displays increased resistance to common road challenges like cracking. The rise in saturates and resins and the control over asphaltene content suggest that WHC-enhanced asphalt roads may offer better durability and extended lifespans. 

### 3.2. Evaluating the Microstructural and Thermodynamic Influence of Waste Honeycombs on AP-5 Bitumen Stability through the Gaestel Index (I_C_)

In the realm of asphalt colloidal science, the pivotal factor for ascertaining the degree of incompatibility or instability between the base AP-5 asphalt and waste honeycomb (WHC)—both pre and post modification/aging—is the Colloidal Instability Index, often referred to as the Gaestel Index (I_C_). This index serves as a reliable barometer illuminating the intricate microstructural nature of the binder.

Historically, bitumen has been characterized as a colloidal system. Within this system, aromatics combined with resins function as stabilizers for asphaltenes. In contrast, saturates paired with asphaltenes act as destabilizing agents. The I_C_ can be mathematically represented as the ratio of the dispersed phase (comprised of flocculants or saturated oils and asphaltenes) to the dispersing phase (which includes peptizers or resins and solvents or aromatic oils) specific to the gas oil, that is, saturates and aromatics [34,35]. It is noteworthy that a lower I_C_ value is indicative of enhanced asphaltene stability within oil. Specifically, I_C_ values equal to or greater than 0.9 imply the precariousness of asphaltenes in an oil matrix, resulting in precipitation or flocculation and forming a feeble, continuous network. Conversely, I_C_ values equal to or below 0.7 suggest that the asphaltene entities exhibit robust stability, meticulously orchestrated by the resins. I_C_ values between 0.7 and 0.9 tread in a gray zone concerning stability [34,35].

**Figure 4:**
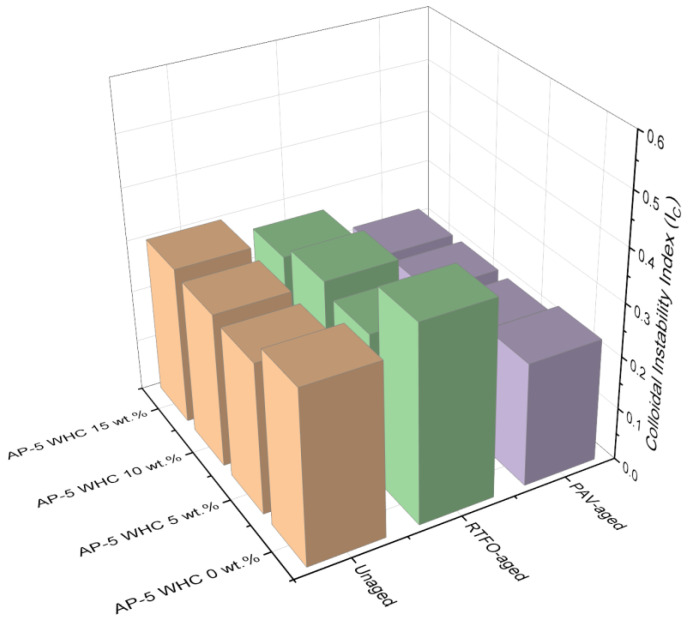
Influence of varied concentrations (5, 10, 15 wt.%) of waste honeycomb (WHC) on the colloidal instability index (I_C_) of AP-5 asphalt prior to and subsequent to RTFO and PAV aging.

Within the purview of the data presented in Figure 4, an astute observation of the AP-5 bitumen’s behavior emerges, particularly when juxtaposed with varying concentrations of WHC. The unadulterated state of AP-5 bitumen, devoid of any WHC infusion (i.e., AP-5 WHC 0 wt.%), indicates an I_C_ metric of 0.3284, a testament to its innate thermodynamic stability.

The compositional constitution of WHC, which is predominantly resinous (92.07 ± 0.89 wt.%) with a minor presence of saturates (7.92 ± 0.89 wt.%), plays a seminal role in modulating the microstructural attributes of the bitumen matrix.

It is imperative to accentuate the manifest decrease in the asphaltene fraction with the progressive incorporation of WHC, a trend consistently observed under both unaging and aging conditions (i.e., RTFO/short term and PAV/long term). Asphaltenes, traditionally attributed with predisposing bituminous materials to instability, witness a quantifiable decrement upon WHC integration, thereby illuminating the transformative role WHC undertakes in reconfiguring bitumen’s equilibrium state.

Concurrently, there are discernible variations in the bitumen’s rheological parameters. A salient decrease is observed in the bitumen’s penetration, ductility, and viscosity profiles with escalating WHC concentrations. Simultaneously, the softening point demonstrates an ascent, indicative of a heightened material stiffness. The abundant presence of beeswax within WHC, renowned for its inherent rigidity, undeniably impinges upon these rheological shifts.

Turning our attention to the aging dynamics, both the RTFO and PAV paradigms amplify the diminution in I_C_ values in tandem with augmenting WHC concentrations. While this trajectory is incontrovertibly influenced by the transmutation of certain naphthenic aromatics into resins [36], it is concomitantly imperative to factor in the transformation of specific asphaltene segments into resins over the temporal spectrum [36]. Such an evolution, accentuated by the resin-rich profile of WHC, amplifies the robust interplay between peptizing agents—predominantly resins—and the asphaltene nucleus. These intricate interactions militate against their potential dissociation from the dispersion milieu, bestowing upon the bitumen–WHC amalgamation an enhanced thermodynamic stability.

In summation, the judicious integration of WHC, with its pronounced resinous constitution, unequivocally emerges as a pivotal modulator in refining both the microstructural and thermodynamic behavior of AP-5 bitumen, as elucidated through diverse physical metrics and I_C_ values across varied aging states.

From a colloidal chemistry vantage point, the bitumen binder is envisioned as a suspensoid. This comprises myriad micelles, each with a resinous exterior and an asphaltenic core, seamlessly dispersed in a continuum of a maltenic phase (a blend of aromatics and saturates) [37]. Depending on the equilibrium dynamics among SARA components, the bituminous binder is typified into three categories: (1) sol-type: primarily liquid in consistency with Newtonian behavior; (2) sol–gel type: exhibiting a balanced solid–liquid consistency with viscoelastic characteristics; and (3) gel-type: dominantly solid with non-Newtonian behavior [38].

(1) Binders categorized as sol-bitumina (I_C_ ≤ 0.7) have a marked presence of resins and oils but a diminished proportion of asphaltenes. Post-cracking, these binders possess the intrinsic ability for self-repair [39]. Both unaged and aged AP-5 asphalt, augmented with varying WHC concentrations, are emblematic of this binder class. During RTFO and PAV aging phases, resin accumulation elevates the stability of suspended asphaltenes, leading to the formation of smaller agglomerates.
(2) Sol–gel bitumina (0.7 < I_C_ < 1.2) are typified by a balanced presence of asphaltene micelles and exhibit a dual elastic–viscous behavior. Their utility in road construction is recognized due to their superior rheological attributes [39].
(3) Lastly, gel-bitumina (I_C_ > 1.2) are distinguished by their well-structured, high-count asphaltene micelles. Their application is best suited for architectural/petroleum asphalts given their distinct “gel” configuration [39].

The empirical reliability of I_C_ as a gauge still necessitates comprehensive practical validation.

### 3.3. Comparative FT-IR Spectroscopy Analysis of AP-5 Asphalt and WHC Modifications: Molecular Alterations and Aging Dynamics

**Figure 5:**
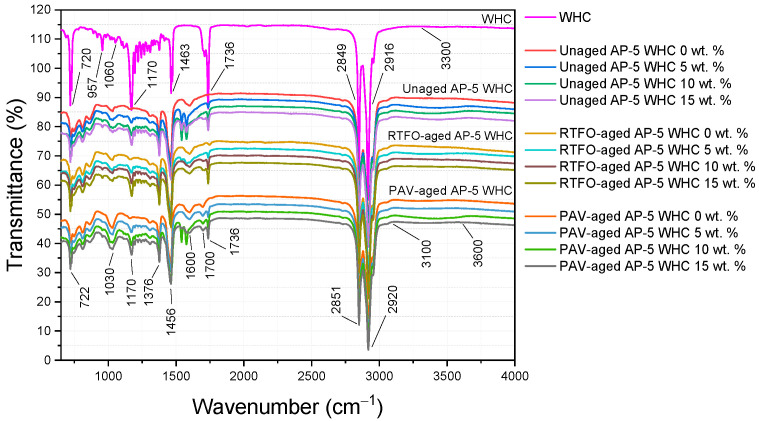
Comparative FT-IR spectra analysis of waste honeycomb (WHC), pristine AP-5 bitumen, and AP-5 bitumen modified with varied concentrations of WHC (5, 10, and 15 wt.%) pre- and post-RTFO and PAV aging processes.

Fourier-transform infrared spectroscopy (FT-IR), a versatile and informative analytical technique, was employed to examine the chemical and molecular alterations in AP-5 asphalt following the integration of various proportions of waste honeycomb (WHC). This examination was conducted before and subsequent to the aging process, furnishing an in-depth understanding of the molecular dynamics. Figure 5 displays a suite of infrared spectra corresponding to the WHC samples along with the unaged, RTFO-, and PAV-aged bitumen.

The unmodified AP-5 asphalt and the WHC infrared spectra share a broad, slightly indistinct peak in the range of 3100~3600 cm^−1^ originating from the bending and stretching excitations of hydroxyl (O–H) and/or amine (N–H) functional groups. Noteworthy peaks at 2920 and 2851 cm^−1^ are representative of the asymmetric and symmetric stretching vibrations of the CH_2_ molecule. Peaks at 1376 cm^−1^ and 1456 cm^−1^ result from C–H asymmetric bending vibrations of –CH_3_ and –(CH_2_)_n_ groups, respectively. The characteristic peak with moderate intensity at 722 cm^−1^ indicates the *in*-plane rocking motion, or bending, of the –CH_2_ molecule. A comprehensive analysis of these signals, chiefly emanating from alkyl functional groups, suggests that the saturated compounds display minimal reactivity towards WHC and thermal processes, thereby attesting to their chemical inertness [40,41].

The characteristic “shoulder” around 1600 cm^−1^, indicative of carbon–carbon stretching vibrations in aromatic rings, can be observed in asphaltic materials. This is a hallmark feature of aromatic substances. Bands appearing at 864, 745, and 548 cm^−1^ can be attributed to the C–H stretches (wags) of para-, meta-, and ortho-substituted benzene rings. These aromaticity-related peaks are visibly influenced by aging and WHC treatment, implying a substantial enrichment of heavier constituents, such as resins and asphaltenes, within the binder [40,41].

The carbonyl (C=O) band, barely discernable in the raw AP-5 bitumen’s infrared spectrogram at approximately 1700 cm^−1^, acquires a heightened visibility after exposure to PAV aging [42,43]. A similar elevation in the intensity of the wide absorption band at 1030 cm^−1^, associated with sulfoxide (S=O) functional groups, is discernable post-RTFO aging [42,43]. This observation underlines the differential impacts of aging on these molecular structures, indicating an increased tendency towards oxidation. The proliferation of oxidation products occurs in tandem with the rise of larger polar compounds, specifically resins and asphaltenes, that significantly influence the viscoelastic solid behavior of asphalt. Consequent to these modifications, the binder’s rigidity escalates, thereby enhancing its susceptibility to fatigue cracking.

The FT-IR spectrum of waste honeycomb (WHC) clearly illustrates the characteristic attributes of beeswax [44,45,46]. The weak broad band observed at approximately 3300 cm^−1^ is attributed to the O–H stretching originating from hydroxyl groups. This particular peak typically signifies the presence of free fatty acids within the sample. In the region of 2916 cm^−1^ and 2849 cm^−1^, the peaks are discerned. These resonances arise due to the C–H stretching found in CH_2_ and CH_3_ groups. Such groups are prominent in the long-chain hydrocarbons as well as the fatty acids and esters that are abundant in beeswax. A distinct peak is detected at 1736 cm^−1^. This strong resonance is indicative of the C=O stretching that is characteristic of esters. It is noteworthy to mention that beeswax encompasses a considerable quantity of wax esters. These esters are essentially the confluence of fatty acids and alcohols. Moving to the peak at 1463 cm^−1^, this is generated due to the bending modes inherent to the CH_2_ and CH_3_ groups present in the compound. In the regions of 1170 cm^−1^ and 1060 cm^−1^, the peaks that emerge are predominantly associated with C–O stretching. This is a clear marker indicating the presence of both esters and alcohols within the material. Lastly, the resonance found at 720 cm^−1^ is attributable to the rocking mode of the CH_2_ groups, further contributing to the detailed spectral signature of beeswax.

In the Fourier-transform infrared (FT-IR) spectroscopic analysis of the honeycomb structure, a prominent peak was identified around 957 cm^−1^. This peak can be ascribed to the C–O–C stretching vibration, which is emblematic of glycosidic linkages [47,48]. Given the inherent composition of honeycomb, which retains sugar residues due to encapsulated honey, this vibration is suggestive of the presence of saccharides, more precisely the β-anomers of glucose residues [47,48]. This observation underscores the presence of polysaccharides within the honeycomb matrix.

In addition to these findings, a distinct pattern of signal intensification was observed for the aforementioned IR absorption peaks following the treatment of pristine AP-5 asphalt with varying dosages of WHC (5, 10, 15 wt.%). This result suggests a change in the chemical composition, although no additional bands are formed in the binder-additive blends, hinting at the predominantly physical nature of the reaction. This assertion provides an insightful perspective on the complex interplay between these materials and their resultant influence on the chemical and physical properties of the asphalt.

### 3.4. Scanning Electron Microscopy (SEM) Characterization: The Intersection of Waste Honeycomb (WHC) Utilization and Bitumen Engineering

A rigorous evaluation was executed to discern the implications of integrating variable concentrations of waste honeycomb (WHC), specifically 5, 10, and 15 wt.%, on the surface topography and underlying microstructure of pristine base AP-5 bitumen leveraging the high-resolution capabilities of scanning electron microscopy (SEM).

**Figure 6:**
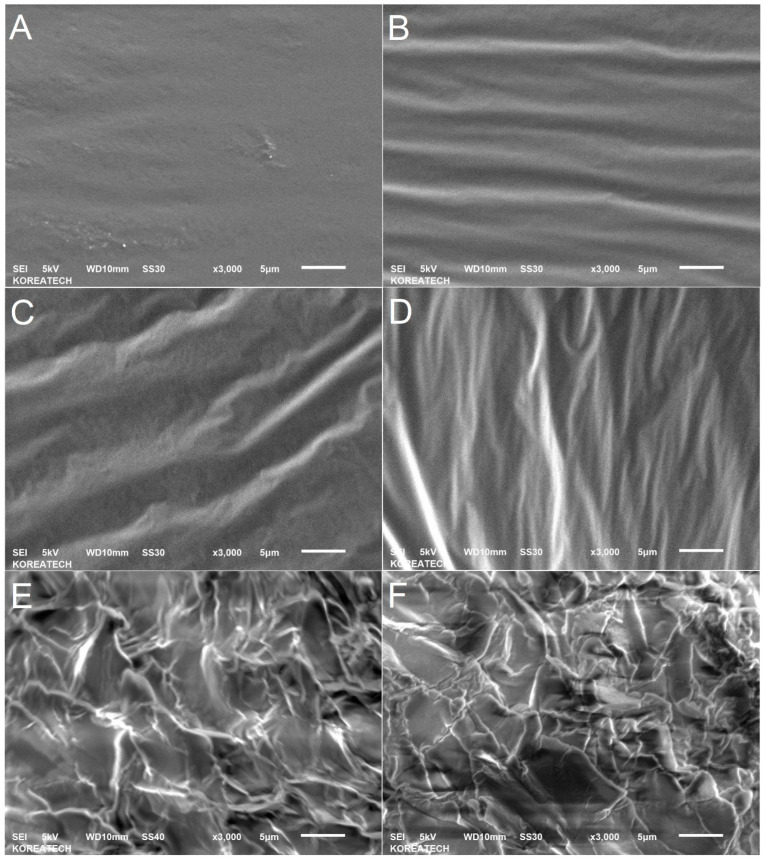
High-resolution scanning electron microscopy (SEM) image of both unmodified and WHC-integrated asphalt specimens alongside pure waste honeycombs (WHCs) captured at a magnification of ×3000. Panels: (**A**) AP-5 with 0 wt.% WHC, (**B**) AP-5 supplemented with 5 wt.% WHC, (**C**) AP-5 containing 10 wt.% WHC, (**D**) AP-5 enriched with 15 wt.% WHC, and (**E**,**F**) the inherent morphology of WHC.

Figure 6A–D elucidates the intricate morphological nuances of both the unmodified AP-5 asphalt (i.e., AP-5 WHC 0 wt.%) and its derivatives with incremental infusions of WHC. With the gradual rise in WHC concentration, a pronounced augmentation in surface roughness is manifest, evoking a conspicuously non-uniform and sinusoidal texture. Such a topographical evolution alludes to an inherent compatibility and optimized integration of WHC within the bituminous milieu, possibly stemming from shared compositional attributes, notably saturates and resins. To fortify this premise of compatibility, deploying complementary techniques, such as fluorescence microscopy, stands as a prudent next step.

Intriguingly, despite the elevated WHC concentrations, SEM depictions refrain from delineating overt honeycomb particulates interspersed within the continuous asphaltic phase, as cataloged in Figure 6B–D. Such an observation begets the conceptualization of a biphasic matrix: the unyielding, continuous asphalt binder and the assimilated WHC forming the discontinuous counterpart. This distinctive phase demarcation not only heralds novel horizons for additive–binder concoctions but also aids in the extraction of pertinent SARA (i.e., saturates, aromatics, resins, and asphaltenes) data via the TLC-FID modality.

Segueing to Figure 6E,F, it presents a vivid depiction of WHC’s inherent morphology. The rough exhibited terrain, which is analogous to parched earth post-hydration evaporation, can be ascribed to the brittleness and rigidity of beeswax at suboptimal temperatures like 25 °C—considerably divergent from its melting point at 64 °C.

Subsequent to methodical evaluation, the strategic deployment of WHC gradients is found to craft superior WHC–binder amalgamations without any discernible compromise in the pivotal engineering traits of the AP-5 asphalt, notably its inherent toughness and resilience. This harmonious blend is predicated on shared physicochemical affinities between the binder and WHC, especially in their structural, polar, and molecular weight dynamics.

In a macroscopic purview, correlating the SEM imprints of WHC–asphalt blends with physical and rheological metrics, one anticipates enhanced road durability and traction. The accentuated surface roughness, underpinned by the WHC integration, might infer roads with augmented longevity and performance metrics, aligning with the optimal balance of flexibility and sturdiness requisite for heavy-duty applications.

Culminating this analysis, it becomes imperative to note that the holistic microstructural and micromorphological outcomes remain inextricably tied to the nuanced interplay between the binder and its additive. This dynamic is inevitably influenced by a myriad of factors, spanning from WHC’s inherent chemical constitution and molecular weight dynamics to the intrinsic traits of bitumen, including its sourcing, gradation, chemical constituents, rheological nuances, and colloidal fabric. 

### 3.5. Thermal Behavior and Stability of WHC-Amalgamated AP-5 Asphalt: Insights from Thermogravimetric Analysis (TGA/DTGA)

**Figure 7:**
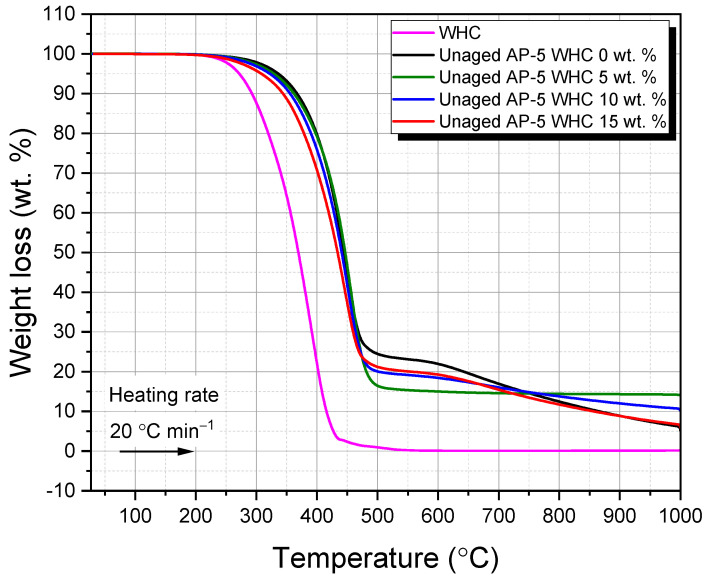
Thermogravimetric analysis (TGA) profiles of waste honeycomb (WHC), pristine AP-5 asphalt, and derivative samples enriched with distinct WHC concentrations (5, 10, and 15 wt.%).

**Figure 8:**
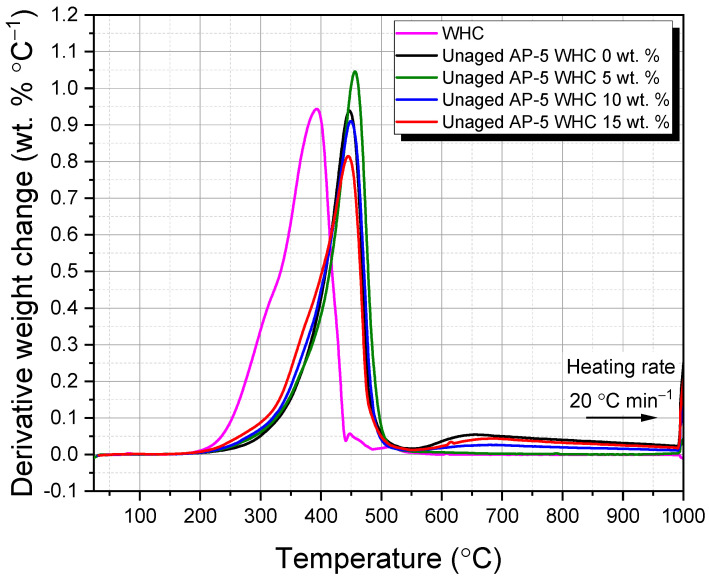
Differential thermogravimetric analysis (DTGA) profiles of waste honeycomb (WHC), untarnished AP-5 asphalt base, and its formulations integrated with distinct WHC concentrations (specifically, 5, 10, and 15 wt.%).

As an astute tool in thermal analysis, thermogravimetric analysis (TGA) was employed to decode the thermal phenomena implicated in the degradation process of waste honeycombs (WHCs) and their amalgamation with AP-5 asphalt. Samples were subjected to a thermal regimen commencing at 25 °C and culminating at 1000 °C, with a controlled increment of 20 °C min^−1^ under a nitrogen atmosphere. The mass loss profile was charted against temperature (TGA/DTGA) to construct primary and derivative thermograms. These graphical depictions were meticulously crafted for WHC, virgin asphalt, and AP-5 asphalt that had been fortified with varying concentrations of WHC (5, 10, and 15 wt.%), as exhibited in Figure 7 and Figure 8.

Figure 7 further demonstrates the thermal influence on the mass of the pristine bitumen (i.e., Unaged AP-5 WHC 0 wt.%). The onset of the major mass loss event at 391.50 °C in the TGA thermogram marks the upper boundary of the binder’s thermal stability. This process unfolds across three distinct thermal regimes: (a) 35.69–391.50, (b) 391.50–470.03, and (c) 470.03–999.95 °C. The initial phase of mass loss (a) can be attributed to the fragmentation and volatilization of saturated and aromatic compounds, accounting for 4.50 and 52.57 wt.% of the binder’s total mass, respectively [49]. The secondary mass loss phase (b), potentially owing to the degradation of a significant fraction of resins and a smaller quantity of asphaltenes, witnesses a complex array of rapid chemical transformations. Aromatic compounds undergo further fragmentation within this thermal window, morphing into more volatile entities [49]. The tertiary phase (c) corresponds to the complete breakdown of asphaltenes, constituting a loss of 18.31 wt.%. The final residue, which emerges post heating to 999.95 °C, comprises approximately 5.06 wt.% carbon. Figure 8 showcases a singular degradation trajectory reaching its zenith at 447.03 °C, a phenomenon presumably engendered by the high concentration of aromatic compounds—constituting 52.57 wt.%—in AP-5 asphalt. This hypothesis gains substantial ground through corroborative evidence garnered via rigorous TLC-FID investigations [49].

WHC’s thermal roadmap signifies three separate stages of weight loss, thereby underscoring its multifaceted thermal composition. The preliminary stage (i), between 35.77 and 317.06 °C, is typified by the expulsion of bound and free water alongside the release of volatile organic compounds such as wax and scent [46]. The subsequent stage (ii), between 317.06 and 422.60 °C, is characterized by one notable mass loss of 99.91 wt.%. This interval is primarily ascribed to the thermal decomposition of resinous constituents present within the beeswax, including long-chain alkanes, acids, esters, polyesters, and hydroxy esters [46]. This is evidenced by one sharp shoulder peaking at 392.76 °C in the DTGA graph (Figure 8). The terminal stage (iii), between 422.60 and 586.43 °C, is governed by the combustion of residual carbon residues resulting from the disintegration of plasticizers, pigments, and additives [46].

Following this, at temperatures exceeding 586.43 °C, virtually all polymeric entities, including mineral and other trace elements, engage in pyrolysis, leaving behind an ash content of approximately 0.20 wt.% at 999.95 °C [46]. The resilience of the WHC under these thermal conditions provides a strong foundation to propose WHC as a potential contender for asphalt modification in road paving applications.

Table 3 elucidates the thermal alterations observed in AP-5 asphalt upon the introduction of WHC. When supplemented with 5 wt.% WHC, the AP-5 asphalt exhibited an enhanced thermal stability, as manifest in the elevated T_onset_ in comparison to its pristine counterpart. This elevation in T_onset_ suggests a potential fortification against temperature-induced variations, which could hypothetically insinuate an extension in the material’s service life.

However, with the incorporation of 10 and 15 wt.% WHC, there is a discernible decrease in the onset degradation temperature. It is imperative to contextualize these data; given that the onset degradation temperature exceeds 300 °C and standard production and application temperatures for asphalt formulations typically do not surpass 200 °C, one must exercise caution in extrapolating real-world implications from this thermal behavior.

Nevertheless, the comprehensive understanding of these thermal characteristics remains paramount. Should subsequent evaluations corroborate that such modifications indeed permit a reduction in production and application temperatures without a commensurate decline in material efficacy, the associated advantages could be manifold. Potential advantages might encompass an attenuation in the binder’s aging trajectory and a contraction in fuel utilization. A diminishment in fuel usage not only harbors economic benefits but also curtails greenhouse gas (GHG) emissive footprints, thereby aligning with sustainability objectives. From an occupational standpoint, attenuated production temperatures might enhance workplace safety by mitigating thermally induced adversities.

In essence, the amalgamation of 5 wt.% WHC appears to bolster the thermal equanimity of AP-5 asphalt. Conversely, the admixture of 10 and 15 wt.% WHC induces thermal alterations that mandate a more meticulous scrutiny, especially when contextualized within the ambit of practical asphalt production and deployment. 

### 3.6. Rigorous Examination of Asphalt Binder Characteristics: An Interplay between Penetration, Softening Point, Viscosity, and Ductility through Waste Honeycomb (WHC) Integration

The systematic exploration conducted aimed to comprehend the sophisticated impact of waste honeycomb (WHC) at varying weight percentages (5, 10, and 15 wt.%) on the rheological behaviors of the AP-5 asphalt binder. This assessment not only covered the unmodified asphalt but also its derivatives post short-term (RTFO) and long-term (PAV) aging treatments. 

#### 3.6.1. Penetration

**Figure 9:**
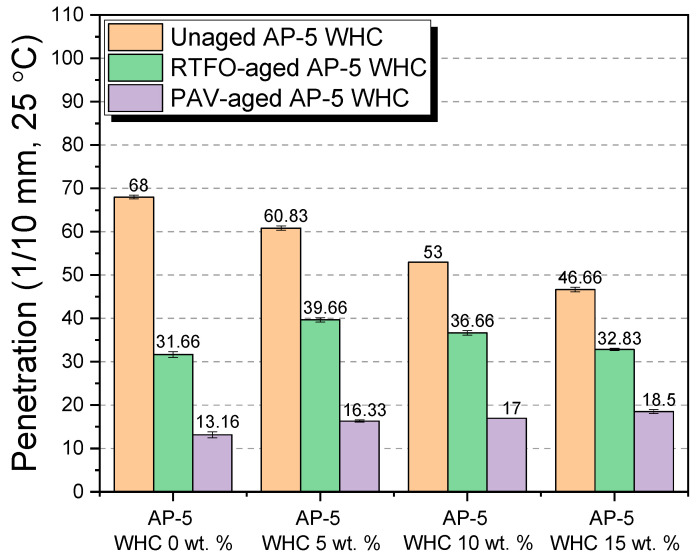
Investigation into the effect of varied waste honeycomb (WHC) concentrations (5, 10, and 15 wt.%) on the penetration attributes of base AP-5 asphalt documented preceding and following the RTFO and PAV aging processes.

Figure 9 provides a comprehensive depiction of the influence exerted by varying concentrations of waste honeycombs—specifically at 5, 10, and 15 wt.% WHC—on the penetration values of AP-5 asphalt. This analysis spans both its pristine state and after the aging processes, including RTFO and PAV.

- Impact on Unaged Asphalt

Upon assessing penetration metrics of unaged asphalt, a significant alteration becomes evident with the introduction of the waste honeycomb (WHC). The penetration value diminishes from an initial 68 ± 0.44 dmm (pertaining to the pure binder) to 46.66 ± 0.51 dmm upon 15 wt.% WHC integration. This change is primarily attributed to WHC’s substantial resin content, approximately 92.07 ± 0.89 wt.%. These polar resins amplify the binder’s rigidity by fostering the conversion of its aromatic compounds into more resins. Additionally, the inherent brittleness of natural beeswax at cooler temperatures, coupled with its melting point ranging between 62 to 64 °C, further accentuates the binder’s stiffness, especially in colder conditions, and offers insight into the modified penetration metrics.

- Short-Term Aging Conditions (RTFO)

The asphalt’s susceptibility to aging processes, especially oxidation, leads to the conversion of its aromatic components into resins, which further transition into asphaltenes [50]. With the introduction of WHC in RTFO-aged conditions, there is an upswing in penetration values, particularly when compared to the properties of unmodified asphalt. This is indicative of the WHC’s capacity to introduce more resins, which peptize and stabilize the asphaltenes in the dispersive-oily medium, thereby reducing the asphalt’s overall hardness.

- Long-Term Aging Conditions (PAV)

Similar to the RTFO condition, the continual addition of WHC in PAV conditions led to a consistent rise in the penetration value. This is attributable to the extended interaction of WHC’s resins with the aged binder, facilitating more effective peptization of asphaltenes, rendering the binder softer and more penetrative over prolonged aging.

- Implications Pertaining to Pavement Performance

From a sophisticated pavement engineering perspective, an examination of the bituminous binder’s rheological properties in the context of waste honeycomb inclusion offers noteworthy insights. The discernible reduction in penetration of the asphalt with incremental admixing of waste honeycombs under pristine conditions suggests a consequential amplification in binder rigidity. Such an elevation in stiffness, while potentially advantageous in some applications, might be counterproductive in colder climates, where an increased brittleness could precipitate premature fatigue cracking.

Conversely, the elevation in penetration following the RTFO and PAV aging procedures signifies a propensity for the binder to soften. Though such a softening could mitigate the risks associated with cold-induced brittleness, prudence is indispensable. A binder that exhibits excessive pliancy might fall short in providing the essential mechanical robustness, heightening susceptibility to deformation, particularly rutting, under substantial vehicular loading in thermally elevated conditions.

In summation, while waste honeycombs demonstrate promise as augmentative agents for bituminous binders, the precise ramifications of their incorporation hinge upon a multitude of variables, from their intrinsic compositional nuances to the temporal state of the binder’s oxidative aging. As such, judicious and empirically guided integration is paramount to capitalize on the prospective benefits without undermining the structural cohesiveness of the pavement.

#### 3.6.2. Softening Point

**Figure 10:**
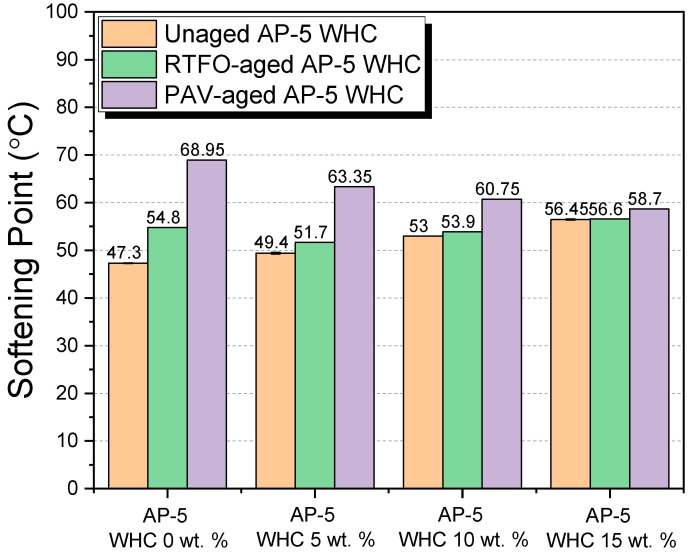
Evaluation of waste honeycomb (WHC) concentrations (namely, 5, 10, and 15 wt.%) on the softening point dynamics of base AP-5 asphalt captured prior to and following RTFO and PAV aging intervals.

Figure 10, drawing upon the ring and ball method, elucidates the influence of waste honeycomb (WHC) on the binder’s softening point (T_R&B_) at high operational temperatures. The binder’s malleability and its propensity to become fluid under temperature stresses are intricately tied to its softening point.

- Impact on Unaged Asphalt

Under fresh binder conditions, introducing WHC to the asphalt matrix leads to an uptick in the softening point value. Specifically, with a progressive increase in WHC from 0 to 15 wt.%, the softening point escalates from 47.30 ± 0.11 °C to 56.45 ± 0.17 °C. Such an increase is largely underpinned by the pronounced presence of resins in WHC, estimated at around 92.07 ± 0.89 wt.%. These resins, as polar components, inherently amplify the binder’s hardness and stiffness, thus raising the softening point. The minor inclusion of saturates, roughly 7.92 ± 0.89 wt.%, might also subtly contribute to this elevation.

- Short-Term Aging Conditions (RTFO)

Aging processes, particularly RTFO, engender shifts within the asphalt’s composition. Aromatics metamorphose into resins, which further transform into asphaltenes [51]—both these phases ramping up the binder’s rigidity [51]. Interestingly, in this aged state, the addition of 5 and 10 wt.% WHC led to a decline in the softening point. Conversely, a 15 wt.% WHC addition presented an increase. This can be attributed to two primary dynamics:

(1) The rejuvenating action of the beeswax components in WHC. Predominantly, the resins in WHC, coupled with its saturated hydrocarbons, esters, free fatty acids, and other compounds, facilitate the peptization of asphaltenes, stabilizing them amidst the maltenes. This interaction potentially reduces the binder’s rigidity, explaining the softening point’s decline.
(2) At 15 wt.% WHC, the collective impact of the added resins might override the rejuvenating effect, especially since the resin content is significant. This results in an elevated softening point.

- Long-Term Aging Conditions (PAV)

In the long-term PAV aging phase, the continuing addition of WHC demonstrates a declining trend in the softening point value. This suggests that the maltenes’ peptizing influence on asphaltenes becomes increasingly dominant, especially when considering the profound resin contribution from WHC.

- Implications on Roadway Performance and Materials Handling

A raised softening point, as observed with 15 wt.% WHC in unaged asphalt, indicates enhanced resilience against thermal cracking, offering potential benefits in self-healing. However, this elevation in softening point might also heighten the risk of rutting during high-temperature episodes. For construction scenarios, a high softening point means that the asphalt mix requires greater energy (i.e., higher temperatures) during manufacturing, transportation, and laying processes. This might pose a challenge in terms of energy consumption and equipment wear.

The melting point of WHC, varying between 62 to 64 °C, is crucial for HMA applications. Below this range, WHC mainly stiffens the binder. Above this threshold, it acts to decrease viscosity. Thus, calibrating the WHC inclusion becomes imperative, aligning the softening point with the peak temperatures roads typically experience, approximating 80 °C.

In summation, while the incorporation of WHC shows promise in modulating asphalt properties, judicious selection, factoring in aging conditions and operational requirements, remains paramount. The data and insights drawn from Figure 10 are critical in steering the optimal use of WHC in asphalt applications, both from a performance and a handling perspective.

#### 3.6.3. Rotational Viscosity

**Figure 11:**
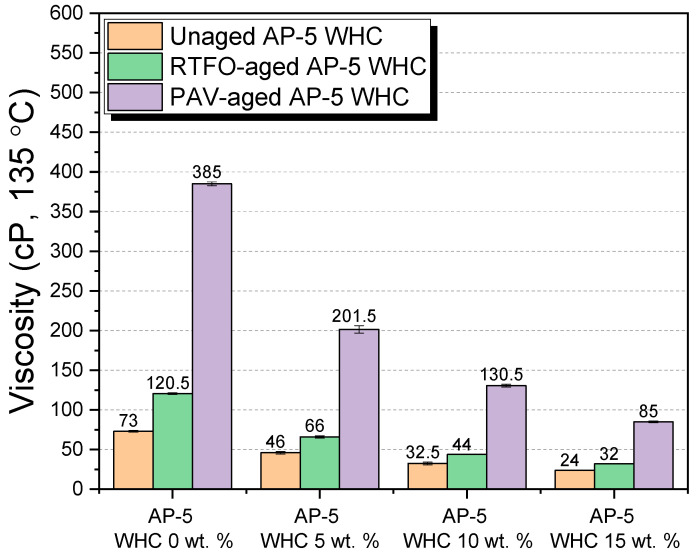
Assessment of varying waste honeycomb (WHC) concentrations (specifically, 5, 10, and 15 wt.%) on the viscosity alterations of base AP-5 asphalt recorded both pre- and post-RTFO and PAV aging phases.

The Brookfield rotational viscosity test, as showcased in Figure 11, was meticulously executed to delineate the flow properties of both unaged and aged asphalt mixtures suffused with different concentrations of WHC. A noteworthy outcome was the systematic diminution in asphalt viscosity upon even distribution of WHC, irrespective of its aging trajectory.

- Impact on Unaged Asphalt

Fresh binder, untouched by any aging effects, demonstrated that the addition of waste honeycomb invariably led to a viscosity reduction. Specifically, a linear decrease was discerned from 73 ± 1.06 cP at 0 wt.% WHC to a mere 24 ± 0.00 cP at 15 wt.% WHC inclusion. Given the constitution of WHC—primarily dominated by resins (approximately 92.07 ± 0.89 wt.%) and, to a minor extent, saturates (approximately 7.92 ± 0.89 wt.%)—this change can be attributed to the resins’ propensity to escalate the fluidity within the binder matrix.

- Short-Term Aging Conditions (RTFO) and Long-Term Aging Conditions (PAV)

The aged asphalt mixtures, subjected to RTFO and PAV, echoed a similar trend, with WHC enrichment resulting in viscosity reduction. However, aging in isolation led to an increase in viscosity across all binders. This aligns with the fundamental understanding of asphalt aging, where aromatics morph into resins and further into asphaltenes [52]. These latter compounds, known for their polar characteristics, amplify the binder’s stiffness and tenacity, thereby escalating its viscosity. The addition of WHC, rich in resins and other components like hydrocarbons, esters, and free fatty acids, rejuvenates aged asphalt. This rejuvenating effect arises from the peptization of asphaltenes, which are rendered more stable in the dispersive medium thanks to the lubrication provided by the maltenes in the binder.

- Implications for Road Performance and Asphalt Handling

The lowered viscosity, particularly evident with a 15 wt.% WHC modification, underscores a heightened workability even at reduced temperatures. This is consequential, as it suggests efficient aggregate coating, translating into improved asphalt mix workability during construction phases such as manufacturing, transport, laying, and compaction. However, there is a caveat. While the augmentation in fluidity can streamline certain construction processes, it can also make the asphalt more susceptible to deformation, possibly causing rutting and bleeding.

Yet, the broader picture presents an auspicious narrative. The use of WHC in asphalt suggests a plethora of benefits:

– Reduction in necessary manufacturing and compaction temperatures, thus mitigating the aging rate.
– Conservation of energy and a corresponding decrease in greenhouse gas emissions.
– Enhanced safety for construction personnel given the diminished necessity for high temperatures.
– Faster road readiness post-construction, enabling earlier vehicular access.

Factoring in the natural composition of beeswax and its source-specific variations, WHC stands out as a promising bio-organic additive, especially for warm-mix asphalt (WMA) technologies. By aligning the benefits of WHC with the unique requirements of each road project, we can harness its full potential in the realm of asphalt rheology.

#### 3.6.4. Ductility

**Figure 12:**
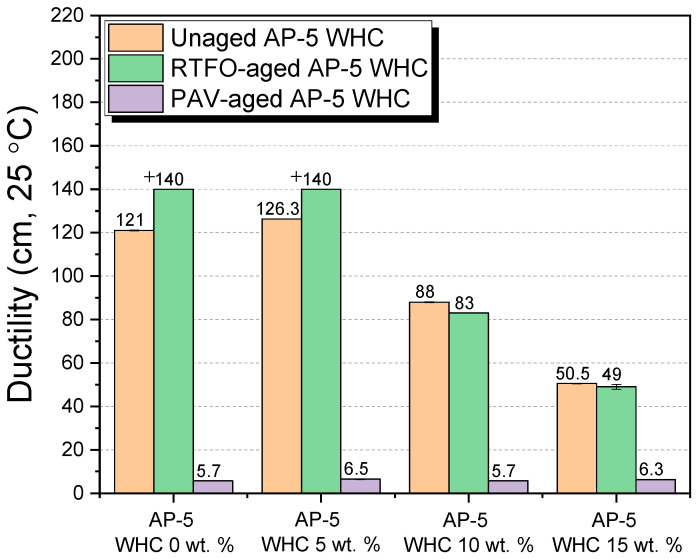
Examination of the impact of distinct waste honeycomb (WHC) concentrations (specifically, 5, 10, and 15 wt.%) on the ductility characteristics of base AP-5 asphalt analyzed prior to and subsequent to RTFO and PAV aging intervals.

In understanding the viscoelastic properties of asphalt binders, particularly ductility, it is pivotal to discern how various additives and aging conditions can influence this parameter. This comprehension directly translates to the asphalt’s practical applicability, performance, and durability on roadways. Referencing Figure 12, we elucidate the influence of waste honeycomb (WHC) on the ductility of base AP-5 asphalt across its fresh, short-term aged (RTFO), and long-term aged (PAV) states.

- Impact on Unaged Asphalt

In its nascent state, the asphalt binder, with a modest 5 wt.% incorporation of WHC, witnessed a significant enhancement in ductility—escalating from an initial 121 ± 0.23 cm to 126.30 ± 0.11 cm. The predominant factor driving this augmentation is the notable resin concentration in WHC, quantified at approximately 92.07 ± 0.89 wt.%. As an integral component of the maltenes, resins confer a peptizing influence on asphaltenes, culminating in enhanced stability within the oleaginous milieu.

However, an intriguing observation emerged when the WHC content is intensified, specifically to thresholds of 10 and 15 wt.%. Contrary to initial trends, these elevated concentrations resulted in a contraction of ductility. The underpinning for this counterintuitive behavior can potentially be rooted in the intrinsic physical properties of beeswax. Recognizing its inherent rigidity at subdued temperatures, the escalating addition of beeswax—as part of the WHC—could impinge upon the ductility profile. Moreover, the multifaceted composition of beeswax might foster diverse interactions that are concentration-dependent, further modulating the ductility metric.

- Short-Term Aging Conditions (RTFO)

The aging trajectory of asphalt is well-understood—the transformation of aromatics into resins, eventually metamorphosing into asphaltenes, augments the binder’s rigidity [53]. Within this context, the introduction of WHC consistently detracts from the ductility. This could be attributed to the delicate equilibrium between the rejuvenating attributes of WHC (like its resin content) and the stiffening tendencies of certain beeswax constituents.

- Long-Term Aging Conditions (PAV)

Contrasting the previous states, WHC’s inclusion within PAV-aged samples does not dramatically alter the ductility. Values remain clustered within the 5.70–6.50 cm range, regardless of WHC’s concentration. This stationary behavior suggests that the peptizing prowess of WHC resins possibly meets a saturation or equilibrium point against the inherent stiffness of PAV-aged binders (I_C 5/10/15 wt.% WHC–AP-5 asphalt_ = 0.21).

- Reflecting on Critical Aspects

The composition of WHC is instrumental in deciphering its interactions with asphalt. While its significant resin content can rejuvenate asphalt by stabilizing the asphaltenes, the multifaceted composition of beeswax—spanning hydrocarbons, esters, free fatty acids, and other compounds—potentially leads to intricate interactions, thereby influencing the ductility variably.

- Concluding Perspective

The introduction of WHC into asphalt binders is a delicate balance of rejuvenation and rheological alteration. While WHC, dominated by its resin content, showcases the potential to bolster ductility under certain conditions, its comprehensive influence, particularly concerning road performance, is contingent on the asphalt’s aging state and the specific WHC concentration. Ensuring this nuanced understanding will be pivotal for harnessing the benefits of WHC in road construction and maintenance processes.

### 3.7. Rutting and Fatigue Cracking Performance of WHC-Infused AP-5 Asphalt: A Comprehensive DSR Analysis

The dynamic shear rheometer (DSR) analysis was meticulously executed to furnish an erudite understanding of the ramifications of varying concentrations of waste honeycomb (WHC), specifically 5, 10, and 15 wt.% WHC, on the rutting susceptibility of both unadulterated and WHC-infused asphalt specimens in both antecedent and subsequent states of RTFO aging. To delve into the intricacies of shear deformation kinetics, we employed the rutting resistance coefficient, articulated as (G*/sin δ). Within this equation, |G*| epitomizes the complex shear modulus, signifying material rigidity, while δ represents the phase angle.

In the realm of rutting resistance, it is sagacious to opt for a binder with elastic and stiff characteristics manifested through elevated magnitudes of (G*/sin δ). Additionally, to curtail the susceptibility to malleability during amalgamation, deployment, and compaction, the stiffness index (G*/sin δ) of nascently synthesized asphalt should surpass 1.0 kPa at the cognate temperature [22]. For RTFO-aged binders, the stiffness index should remain above 2.2 kPa across the mean 7-day maximum pavement design temperature, playing an instrumental role in curtailing rut manifestation [22]. 

**Figure 13:**
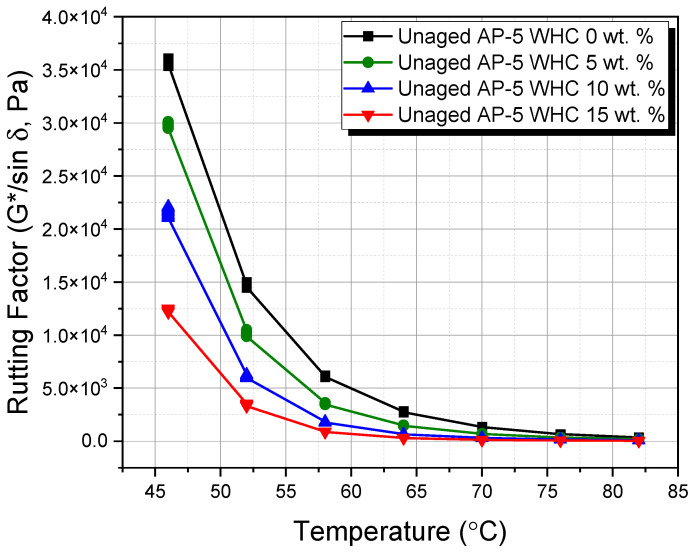
Rutting factor (G*/sin δ) vs. temperature for pristine unaged AP-5 asphalt and specimens with varying concentrations (5, 10, and 15 wt.% WHC) of waste honeycombs.

**Figure 14:**
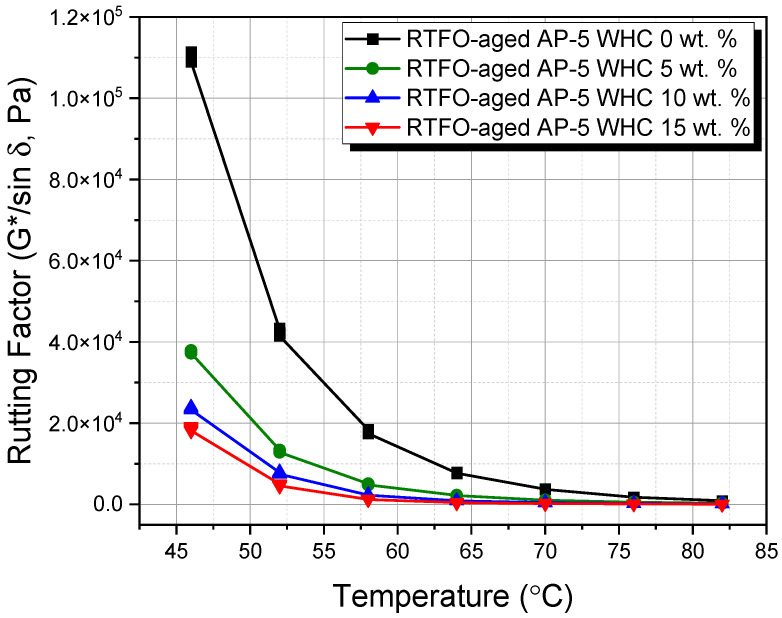
Rutting factor (G*/sin δ) in relation to temperature for RTFO-aged AP-5 asphalt benchmark and derivative specimens integrated with waste honeycombs at concentrations of 5, 10, and 15 wt.% WHC.

Figure 13 and Figure 14 delineate the rutting factor’s distinct vulnerability under non-conditioned scenarios and during RTFO conditioning at elevated thermal ranges, respectively.

In the rigorous assessment, the unadulterated control specimen, AP-5 at 0 wt.% WHC, demonstrated unparalleled resistance to rutting, distinctly overshadowing its WHC-augmented counterparts, as illustrated in Figure 13. Precise analyses through the DSR method discerned a significant shift in the relationship between the rutting factor and temperature subsequent to the integration of waste honeycombs, implying diminished endurance to repetitive stress.

However, an intriguing dichotomy emerges from the dataset. While the rutting factor suggests a potential compromise in performance with WHC integration, penetration test outcomes convey an opposing narrative, revealing augmented rigidity within the binder matrix. This contrast can be systematically attributed to the pronounced beeswax concentration inherent in waste honeycombs. Known for its transition to a rigid and solidified state at ambient temperatures—especially below the melting range of 62 to 64 °C—beeswax elucidates the binder’s reduced rutting factor with increasing test temperatures.

An essential revelation is that at temperatures below 52 °C, all the analytically probed binders fulfilled the sine qua non of the Superpave specifications (G*/sin δ ≥ 1 kPa) [22]. Specifically, the control asphalt and its variants, fortified with judicious amounts of waste honeycomb, seamlessly met this benchmark at temperatures of 70, 64, 64, and 58 °C, respectively.

Figure 14, a graphical elucidation, demystifies the direct repercussion of WHC augmentation on the rutting coefficient of RTFO-aged bituminous samples in the context of temperature. A concordant decline in rutting performance was witnessed, signaling diminished structural rigidity and stability at escalated temperatures. This receding shear deformation yield is attributable to an interplay between increased resin content and waning asphaltene fractions in the asphalt matrix.

It is imperative to highlight that the RTFO-aged binders, imbued with diverse levels of waste honeycomb, withstood rutting until temperatures of 70, 64, 58, and 52 °C, respectively (G*/sin δ ≥ 2.2 kPa) [22]. This persistent wane in the rutting index is emblematic of the deleterious consequences of WHC on thermally induced permanent deformation. A surfeit of WHC could vitiate the binder’s intrinsic capacity to combat thermal deformation during torrid seasons.

Further, an assortment of WHC–bitumen concoctions were subjected to long-term aging (PAV) to scrutinize their thermo-mechanical fidelity and fatigue cracking resilience utilizing the DSR modality. The fatigue cracking coefficient (G*.sin δ) was employed as a yardstick. A lower value for this metric portends augmented resistance to fatigue cracking, while the converse holds true as well. To forestall fatigue-induced distress, it is advisable to utilize a binder with enhanced elasticity and diminished stiffness.

Following exposure to both short-term (RTFO) and long-term aging (PAV), the binder’s stiffness should not breach the threshold of 5000 kPa at a given intermediate temperature, approximating to +4 °C and capped by the extremities of the pavement design temperatures [23]. Straining the pavement beyond 5000 kPa could culminate in fatigue cracking [23].

**Figure 15:**
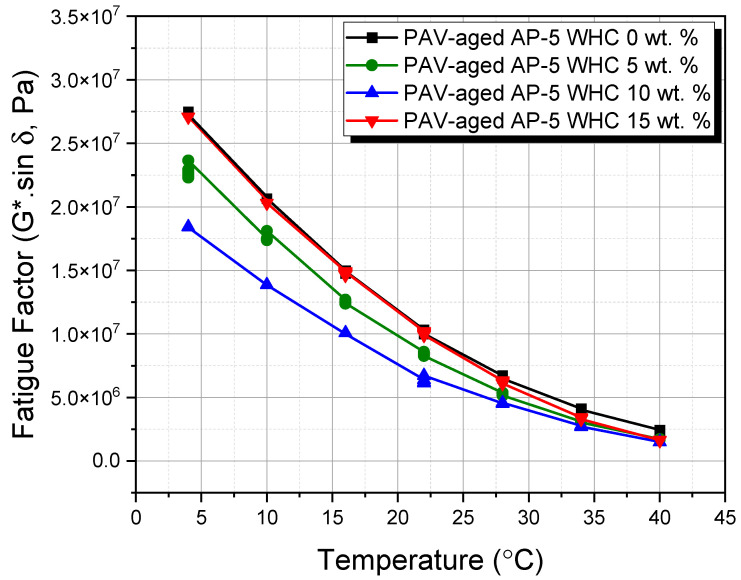
Fatigue cracking factor (G*.sin δ) in correspondence with temperature for PAV-aged AP-5 asphalt benchmark and associated specimens incorporating waste honeycombs at concentrations of 5, 10, and 15 wt.% WHC.

As visually evident in Figure 15, various concentrations of WHC in the base AP-5 asphalt triggered a discernible decrease in the fatigue cracking index (G*.sin δ). Reduced (G*.sin δ) readings signify restrained dissipation of shearing energy, hence conferring enhanced resilience to cracking strains, courtesy of the binder’s resinous compound abundance. All PAV+RTFO-treated asphalt mixtures, regardless of the WHC content, were able to comply with the Superpave requisites at intermediate temperatures of 34, 28, 25, and 25 °C (G*.sin δ ≤ 5000 kPa) [23].

Practically, it seems viable that the pavement’s resistance to cracking can be substantially bolstered by integrating an optimal quantity of discarded honeycombs (below 5 wt.% WHC) with the base AP-5 asphalt.

### 3.8. Deciphering Waste Honeycomb’s Dual Impact: A Superpave-Guided Exploration into the Thermal Performance Spectrum of AP-5 Asphalt Binders

Capitalizing on the Superpave performance classification methodology [54], the dynamic shear rheometer’s empirical evidence was employed for the systematic evaluation of the performance grade (PG) of our foundational AP-5 bitumen and its cohorts, infused with differing proportions of waste honeycombs (WHC)—specifically, 5, 10, and 15 wt.% WHC.

**Figure 16:**
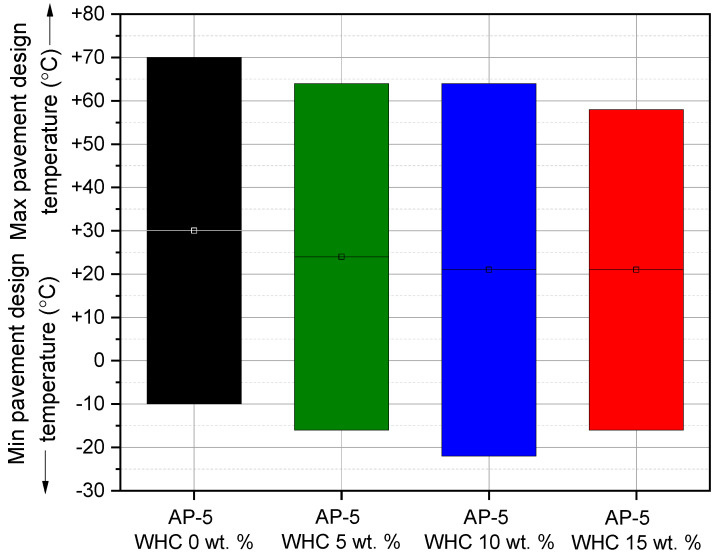
Influence of different waste honeycomb (WHC) concentrations (5, 10, and 15 wt.%) on the performance grade (PG) of AP-5 base asphalt.

Figure 16 elucidates the WHC’s quantifiable impact on the PG metrics of the asphalt binder. The PG 70–10 designation suggests that the pristine form of AP-5 asphalt exhibits robust resilience under conventional traffic strains across temperature variabilities between −10 to 70 °C. The PG system, holistically, delineates the climatic operating boundaries best suited for a selected binder, establishing parameters for both chilling and warming thermal behaviors of bitumen, which are critically associated with road paving performance.

In the context of the unadulterated bitumen, the WHC influence seems unfavorable at escalated temperatures, with the data signifying that every incremental 5 wt.% of the additive was correlated with a grade decrement of +1 (a grade difference corresponds to a six-degree Celsius shift). Nevertheless, this may not compromise the pavement’s structural integrity against profound permanent deformation under elevated temperatures. Conversely, the waste honeycomb expanded the PG variably at more frigid temperatures. In a declining sequence, the additive contributed to grade elevations of +1, +3, and +1, corresponding to mixtures containing 5, 10, and 15 wt.% WHC, respectively. This observation substantiates that the amended binders underwent substantial rigidification, and their predisposition to thermal cracking was effectively subdued at cooler temperatures.

In summation, the investigation underlines that while waste honeycombs may not significantly ameliorate the bitumen’s high-temperature performance in combating rutting distress during warmer seasons, they could substantially bolster the low-temperature performance grade, endowing the pavement with a commendable anti-cracking attribute during the winter periods.

### 3.9. Modulating Rutting Resistance in AP-5 Asphalt with Waste Honeycomb Incorporation: A Dynamic Rheological Assessment via MSCR

The multiple stress creep recovery (MSCR) procedure, established to accurately replicate real-world loading conditions for road infrastructure, was undertaken at elevated temperatures. The primary intent was to rigorously discern the elastic properties of the foundational AP-5 asphalt and its consequent interplay between stress and strength factors. In this context, the ramifications of various concentrations of waste honeycombs (5, 10, and 15 wt.% WHC) on the rutting characteristics of temporally aged bitumen were systematically analyzed using the dynamic shear rheometer (DSR) from Thermo Fisher.

**Figure 17:**
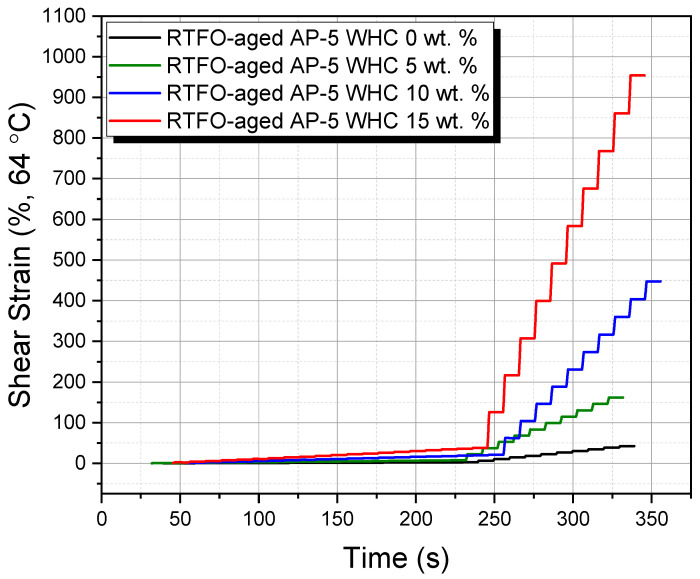
Outcomes of the multiple stress creep recovery (MSCR) assessment at 0.1 kPa, 3.2 kPa, and 64 °C for RTFO-seasoned base AP-5 asphalt alongside its derivatives, inclusive of discrete WHC proportions (namely, 5, 10, and 15 wt.%).

Data encapsulated in Figure 17 delineate MSCR readings at two distinct pressures: 0.1 and 3.2 kPa. Specifically, 0.1 kPa characterizes the bitumen’s performance within the linear viscoelastic spectrum, whilst 3.2 kPa highlights its behavior beyond this threshold, venturing into the non-linear domain [55]. 

Prevailing standards indicate that an RTFO-aged binder, marked by a higher proportion of recoverable strains (*R%*), inherently possesses superior anti-rutting capabilities [56]. Relative to the WHC-augmented asphalt samples, the standard bitumen (specifically, RTFO-aged AP-5 WHC 0 wt.%) recorded the most pronounced *R%* value. Such an observation intimates that the systematic introduction of this organic constituent might not unequivocally enhance the resilience of bituminous aggregates to sustained deformations, potentially compromising their efficacy at high temperatures.

Upon examination of the data presented in Table 4, it is evident that all formulated mixtures exhibited augmented *J_nr_* (i.e., non-recoverable creep compliance) values, notably when juxtaposed with the unmodified binder. This suggests the propensity for rutting as a potential concern for asphaltic formulations comprising higher WHC concentrations. Referencing the AASHTO M 332 Standard Specification [57], the synergy between the MSCR assessment and *J*_*nr* 3.2_ was harnessed to intricately determine bitumen’s rutting resistance. The stipulations are as follows: for exceptionally dense traffic (E), the value of *J_nr_*_3.2_ should not exceed 0.5 kPa^−1^; for significantly danse traffic (V), the threshold is set at 1.0 kPa^−1^; for dense traffic (H), the value is capped at 2.0 kPa^−1^; and for conventional/standard traffic (S), the limit is established at 4.0 kPa^−1^ [57].

Perusing the tabulated data in Table 4, the undiluted asphalt blend (i.e., RTFO-aged AP-5 WHC 0 wt.%) evidently aligns with the robust (H) traffic loading bracket. Conversely, the assorted WHC-infused variants align predominantly with the non-standard (NS) traffic loading, especially when contending with rutting challenges at 64 °C. It is worth noting that while the Superpave performance grading (PG) predominantly focuses on the *J_nr_* parameter at 3.2 kPa, a shear stress magnitude of 1.0 kPa remains critically pertinent, especially when aiming to preserve certain sensitive binders against alterations in volume and shear stress. In alignment with this, the AASHTO M 332 [57] postulates a pivotal criterion, mandating that the deviation in *J_nr_* values between 0.1 and 3.2 kPa remains under the threshold of 75%.

Drawing on the findings delineated in Table 4, each of the asphalt formulations showcased marked compliance with established benchmarks, illustrating commendable stability in the face of shear stress fluctuations. 

Starting with the AP-5 binder devoid of any WHC addition (i.e., AP-5 WHC 0 wt.%), it showcased the lowest Δ*J_nr_* value of 9.60%, signifying its robustness in relation to shear stress variations. Its performance sets a baseline for evaluating the impact of WHC incorporation.

The blend enhanced with 5 wt.% WHC displayed a Δ*J_nr_* value of 22.00%. Although this is an increase in comparison to the pure AP-5 binder, it remains moderate, suggesting that judicious inclusions of WHC, such as this, can still maintain relative stability. The bituminous aggregate’s response to shear stress changes does not exhibit pronounced disruptions even with this WHC percentage, ensuring that under challenging thermal or loading conditions, its structural integrity remains largely preserved.

Progressing to higher WHC concentrations, the AP-5 WHC 10 wt.% formulation registered a further elevation in its Δ*J_nr_*, reaching 30.70%. While the resilience to strain remains commendable, there is an evident trend signifying increasing susceptibility to strain deviations with incremental WHC.

Most noteworthy was the AP-5 WHC 15 wt.% mixture. It demonstrated the most pronounced Δ*J_nr_* value at 46.30%. This conspicuous escalation suggests that elevated concentrations of WHC start to exert a more profound influence on the binder’s structural behavior, potentially heightening its vulnerability to sustained deformations under varying stress conditions.

In essence, while conservative WHC additions like 5 wt.% showcase relative stability, escalating the concentration to 15 wt.% amplifies the strain dynamics, necessitating meticulous considerations for practical applications.

At the stipulated operational temperature of 64 °C, Figure 17 delineates the evolutionary progression of strain accumulation (%) as a function of elapsed time (s). A nuanced dissection of the presented data accentuates the salient modulation induced by the WHC modifier within the realms of recovery and creep mechanics.

As elucidated earlier in Table 4 and reaffirmed by Figure 17, the sequential augmentation of WHC within the asphalt binder engenders a discernible amplification in strain accrual following successive stress–relaxation cycles. Such a revelation accentuates an incontrovertible thesis: while judicious admixtures of WHC can enhance specific mechanical properties, excessive inclusion risks attenuating the asphalt’s intrinsic fortitude against enduring deformative phenomena.

## 4. Conclusions

In this research, we present a seminal progression at the nexus of innovative waste management techniques and sustainable civil engineering practices. This study undertakes a meticulous assessment of the feasibility of waste honeycomb (WHC) as a potent modifier for asphalt binder formulations. By addressing this oft-overlooked apicultural byproduct, we propose an integrated strategy that accentuates both ecological preservation and the fortification of infrastructural robustness.

Employing rigorous methodological standards, the investigation delved into the impact of varying concentrations of WHC (5, 10, and 15 wt.%) on the asphalt binder. The comprehensive battery of both advanced and conventional tests, including TLC-FID, FT-IR, SEM, penetration, and softening point, among others, facilitated an unparalleled depth of insight into the binder’s behavior across different aging scenarios. Central to the findings was the transformative influence of WHC’s robust resinous content (92 ± 07 wt.%) complemented by saturates (7.92 ± 0.89 wt.%). This distinct composition led to marked shifts within the SARA profile, particularly with the significant reduction of asphaltenes, known determinants of binder tenacity.

Further elucidating these insights, the colloidal instability index (I_C_) witnessed a notable decrease across all aging states. This reduction, enhanced by WHC’s inherent properties, affirms a transition towards a more thermodynamically harmonious asphalt matrix. Concurrently, FT-IR diagnostic examinations highlighted a non-chemical, physical interaction between WHC and asphalt, with TGA evaluations underscoring the augmented thermal resilience of the asphalt formulated with a 5 wt.% WHC admixture. SEM interpretations further revealed the homogeneous integration of WHC within the asphalt matrix, hinting at an optimized microstructure.

On examining traditional test outcomes, penetration exhibited variability contingent on aging conditions, while the softening point demonstrated WHC-concentration-dependent dynamics. Viscosity displayed a consistent decrease across scenarios, whereas ductility presented varying behaviors based on WHC concentration and aging modality. From a rheological standpoint, while the introduction of WHC subtly amplified rutting potential, a counterbalancing enhancement in fatigue resistance was observed upon judicious WHC inclusion.

Drawing from the amassed evidence, it can be posited that a 5 wt.% WHC concentration emerges as a potential sweet spot for optimizing asphalt performance. However, the necessity for additional in-depth research is underscored to fortify this proposition and delineate its broader applicability

In summation, this pioneering research unequivocally advocates for the integration of waste honeycomb into asphalt formulations. By amalgamating enhanced road performance with sustainable waste management, the research sets a new benchmark in sustainable civil engineering. These findings underscore the transformative role of WHC, illuminating its promise for future infrastructural endeavors. As the study concludes, it becomes imperative to delve deeper, exploring varied WHC concentrations, long-term performance metrics, and the broader implications for a globally sustainable road infrastructure.

## Figures and Tables

**Table 1:** Chemical-physical characteristics of waste honeycomb (WHC).

Elemental Analysis	Mean ± SD
C (Carbon)	82.15 ± 0.20 wt.%
H (Hydrogen)	14.09 ± 0.04 wt.%
N (Nitrogen)	0.02 ± 0.02 wt.%
S (Sulfur)	0.00 ± 0.00 wt.%
O (Oxygen)	3.92 ± 0.02 wt.%
**SARA Generic Fractions**	
Saturates	7.92 ± 0.89 wt.%
Aromatics	0.00 ± 0.00 wt.%
Resins	92.07 ± 0.89 wt.%
Asphaltenes	0.00 ± 0.00 wt.%
**Physical Properties**	
Softening point/slip melting point	34 ± 0.00 °C
Rotational viscosity at 100 °C	4.70 ± 0.00 cP
Melting point	64 °C
Solubility	Soluble in organic solvents ^†^
Density at 25 °C	0.9500 g cm^−3^
Color	Golden hue
Odor	Sweet–Mild aroma

^†^ Organic solvents: *n*-hexane, toluene, and dichloromethane, among others.

**Table 2:** Physicochemical properties of base AP-5 asphalt cement.

Elemental Analysis	Mean ± SD
C (Carbon)	86.62 ± 1.62 wt.%
H (Hydrogen)	10.78 ± 0.19 wt.%
N (Nitrogen)	0.51 ± 0.02 wt.%
S (Sulfur)	5.65 ± 0.05 wt.%
O (Oxygen)	0.90 ± 0.03 wt.%
**SARA Generic Fractions**	
Saturates	4.50 ± 1.32 wt.%
Aromatics	52.57 ± 2.58 wt.%
Resins	22.70 ± 2.18 wt.%
Asphaltenes	20.22 ± 2.40 wt.%
Colloidal Instability Index (I_C_) **	0.3284
**Physical Properties**	
Penetration at 25 °C	68.00 ± 0.44 dmm
Softening point	47.30 ± 0.11 °C
Rotational viscosity at 135 °C	73.00 ± 1.06 cP
Ductility at 25 °C	121.00 ± 0.23 cm
Density at 25 °C	1.00 ± 00 g cm^−3^

** I_C_ = ([Saturates] + [Asphaltenes])/([Aromatics] + [Resins]).

**Table 3:** TGA/DTGA thermogram data: comparative analysis of waste honeycombs (WHCs), unprocessed AP-5 asphalt base, and WHC–AP-5 composite blends with respective loadings of 5, 10, and 15 wt.% WHC evaluated at a controlled heating rate of 20 °C min^−1^.

Sample	TGA/DTGA (°C)						−ΔW (wt.%)
	Stage 1	Stage 2	Stage 3	T_onset_	T_offset_	T_max_	
WHC	35.77~317.06	317.06~422.60	422.60~999.95	317.06	422.60	392.76	0.20
AP-5 WHC 0 wt.%	35.69~391.50	391.50~470.03	470.03~999.95	391.50	470.03	447.03	5.06
AP-5 WHC 5 wt.%	24.07~399.54	399.54~479.75	479.75~999.95	399.54	479.75	456.27	13.96
AP-5 WHC 10 wt.%	30.24~386.69	386.69~472.05	472.05~999.97	386.69	472.05	449.79	10.06
AP-5 WHC 15 wt.%	32.00~373.35	373.35~467.24	467.24~999.96	373.35	467.24	445.16	5.76

T_onset_: Initiation temperature for thermal degradation (°C); T_offset_: Termination temperature corresponding to final weight loss (°C); T_max_: Peak decomposition temperature (°C); ΔW: Residual carbonaceous content measured at 1000 °C (wt.%).

**Table 4:** Derivative parameters from the multiple stress creep recovery (MSCR) evaluation at 0.1 kPa, 3.2 kPa, and 64 °C for RTFO-conditioned base AP-5 asphalt and its specimens incorporating distinct proportions of waste honeycombs (5, 10, and 15 wt.% WHC).

Asphalt Binder Type	MSCR Data at 64 °C					
	*R* _0.1_ (%)	*R* _3.2_ (%)	*J*_*nr* 0.1_ (kPa^−1^)	*J*_*nr* 3.2_ (kPa^−1^)	Δ*J_nr_* (%)	PG Grade
AP-5 WHC 0 wt.%	−2.20	−7.60	1.1407	1.2499	9.60	PG 64 H
AP-5 WHC 5 wt.%	−5.30	−10.00	3.9502	4.8193	22.00	PG 64 NS
AP-5 WHC 10 wt.%	−3.80	−10.90	10.2050	13.3342	30.70	PG 64 NS
AP-5 WHC 15 wt.%	−4.60	−11.80	19.5714	28.6285	46.30	PG 64 NS

H: Heavy traffic loading; NS: Non-standard/unspecified traffic loading.

## Data Availability

Data from this study can be accessed upon request by reaching out to the first author.
